# The Transcriptomic Analysis of Circulating Immune Cells in a Celiac Family Unveils Further Insights Into Disease Pathogenesis

**DOI:** 10.3389/fmed.2018.00182

**Published:** 2018-06-19

**Authors:** Rachele Ciccocioppo, Simona Panelli, Maria C. Conti Bellocchi, Giuseppina C. Cangemi, Luca Frulloni, Enrica Capelli, Gino R. Corazza

**Affiliations:** ^1^Gastroenterology Unit, Department of Medicine, Azienda Ospedaliera Universitaria Integrata Verona, University of Verona Verona, Italy; ^2^Pediatric Clinical Research Center “Invernizzi”, University of Milano Milan, Italy; ^3^Clinica Medica I, Department of Internal Medicine, Fondazione IRCCS Policlinico San Matteo, University of Pavia Pavia, Italy; ^4^Department of Earth and Environmental Sciences, University of Pavia Pavia, Italy

**Keywords:** celiac disease, exosome and immune modulation, microarray analysis, pathogenesis, transcription factors

## Abstract

Celiac disease (CD), the most common chronic enteropathy worldwide, is triggered and sustained by a dysregulated immune response to dietary gluten in genetically susceptible individuals. Up to date either the role of environmental factors and the pathways leading to mucosal damage have been only partially unraveled. Therefore, we seized the unique opportunity to study a naturally-occurring experimental model of a family composed of both parents suffering from CD (one on a gluten-free diet) and two non-celiac daughters. The control group consisted in four unrelated cases, two celiac and two non-celiac subjects, all matching with family members for both disease status and genetic susceptibility. In this privileged setting, we sought to investigate gene expression in peripheral blood mononuclear cells (PBMCs), a population known to mirror the immune response state within the gut. To this purpose, PBMCs were obtained from the four biopsied-proven CD patients and the four non-celiac cases. Each group included two family members and two unrelated control subjects. After RNA purification and cDNA synthesis, each sample underwent a microarray screen on a whole-transcriptome scale, and the hybridization results were visualized by hierarchical clustering. Differentially expressed genes (DEG) were partitioned into clusters displaying comparable regulations among samples. These clusters were subjected to both functional and pathway analysis by using the Kyoto Encyclopedia of Genes and Genomes. Interestingly, on a global gene expression level, the family members clustered together, regardless of their disease status. A relevant fraction of DEG belonged to a limited number of pathways, and could be differentiated based on disease status: active CD vs. treated CD and CD vs. controls. These pathways were mainly involved in immune function regulation, cell-cell junctions, protein targeting and degradation, exosome trafficking, and signal transduction. Worth of noting, a small group of genes mapping on the male-specific region of the Y chromosome, and previously linked to cardiovascular risk, was found to be strongly upregulated in the active CD case belonging to the family, who suddenly died of a heart attack. Our results provide novel information on CD pathogenesis and may be useful in identifying new therapeutic tools and risk factors associated with this condition.

## Introduction

Celiac disease (CD) is a high prevalent multifactorial disorder caused by a dysregulated immune response toward both dietary (i.e., the gluten components) and self (i.e., the enzyme tissue transglutaminase) antigens: it has the small intestine as the target organ and develops in genetically susceptible individuals (1). Its clinical picture ranges from an asymptomatic or oligosymptomatic condition to a severe malabsorption syndrome, and its treatment is still based on a gluten-free diet (GFD), as originally proposed by the Dutch Pediatrician, Doctor Willem Dicke (2). It is widely accepted that genetic factors play a crucial role, as supported by the familial occurrence of the disease, with first-degree relatives of patients presenting with an incidence of about 15%:10–20 times higher than the general population (3). Concordance in dizygotic twins is 10–20% and reaches 70–80% in monozygotic twins (4, 5). The main genetic susceptibility factor is represented by the genomic region known as “human leukocyte antigen” (HLA) on chromosome 6 (6p21). This region is also known as “locus celiac 1.” Specifically, CD is strongly associated with two HLA class II haplotypes (6). Indeed, more than 90% of patients carry the DQ2 heterodimer, coded by the α chain alleles DQA1 ^*^0501 or ^*^0505, and β chain alleles DQB1 ^*^0201 or ^*^0202. The remaining carry the DQ8 heterodimer, coded by DQA1 ^*^0301 and DQB1 ^*^0302 alleles, while only a few patients carry the DQB1 risk allele (7). Both heterodimers are expressed on antigen-presenting cells and are responsible for the high affinity to gluten peptides and their efficient presentation to T-cells (7). An additional 40 non-HLA susceptibility loci have been identified (8, 9). Among these, loci “celiac 2” on chromosome 5 (5q31-q33), “celiac 3” on chromosome 2 (2q33), and “celiac 4” on chromosome 19 (19p13.1) (10–12). However, epidemiological data suggest that further predisposing loci might exist (13). Indeed, 39% of the general population carries the HLA DQ2 or DQ8 haplotypes, but only 3% of them will eventually develop CD. Recent studies point to the involvement of non-coding regions with their role in gene expression regulation(14). Specific gene networks, as well as environmental factors, could also contribute to mucosal lesions (15). In other words, considering the extent and complexity of the CD-specific dysregulated immune response, every new contribution aimed at discovering still unidentified genes, pathways, or factors linked to (or differentially expressed in) this condition, may help to clarify pathogenic routes.

This study aimed to uncover new biological pathways involved in CD pathogenesis through a whole-transcriptome study on peripheral blood mononuclear cells (PBMCs). Blood was sampled from a naturally-occurring experimental setting of a family composed of both parents suffering from CD (one on a gluten-free diet) and two non-celiac daughters. A control group was included in the study that consisted in four disease-matched unrelated subjects: two celiac patients and two non-celiac subjects, all carrying the classic genetic susceptibility, in an attempt to identify further risk factors involved in disease onset. PBMC appeared particularly suitable for this study for several reasons. They can be harvested in a non-invasive fashion and a wealth of published data report a continuous crosstalk between immune cells and other compartments of the body, comprised the digestive tract (16–18). Furthermore, PBMCs include antigen-specific dendritic cells, T- and B-lymphocytes which, apart from being directly responsible for the tissue damage in the small intestine, are known to reflect events in the target organ by recirculating from the intestinal mucosa to peripheral blood (17). For example, a set of genes with dysregulated expression in PBMCs has been recently defined in pediatric CD (16), with a good discriminating potential between patients and controls and possible diagnostic applications. In general, peripheral blood is known to be a vehicle for transfer of molecular signals (19, 20). This exchange is mediated primarily by exosomes, which contain and transfer signals represented by DNA, mRNA, microRNA, and proteins. On one hand, this has the potential of modifying the gene expression picture of acceptor cells, where mRNA incorporated into exosomes may be translated and microRNA exert their regulatory role (21). On the other hand, this exchange mediated by peripheral blood informs the whole body about the eventual disease status of an organ (19). Strictly related to this, it is known that gut epithelium and immune cells, when within the gut, contain (and then release) exosome-like vesicles that carry HLA class II/antigen complexes, including food antigens (22, 23).

For all the reasons outlined above, the experimental setting and PBMC samples provided us with a privileged view to better understand the influence, and relative weight, of genetic relatedness and environment in this complex, multifactorial disorder.

## Methods

### Experimental cohort

All celiac patients included in the study have been diagnosed according to the worldwide accepted criteria (i.e., positivity for anti-tissue transglutaminase class A autoantibodies and presence of characteristic mucosal lesions upon histology of the duodenal mucosa) (24).

The demographic and clinical features of the eight total patients and controls are listed in Table 1. In the table, and hereafter in the text, each case is indicated as “cc” followed by a number. Subjects cc1–cc4 constitute the family. Subjects cc5–cc8 are unrelated individuals, external to the family.

**Table 1:** Patients and controls included in the study.

**Sample code**	**Date of birth/sex**	**Diagnosis-relatedness/unrelatedness**	**Position within the family**	**Body mass index**	**Comorbidities**
cc1	29-06-1964/F	TCD-r	Mother	21.1	Miscarriage Osteopenia
cc2	16-11-1960/M	UCD-r	Father	23.0	*Helicobacter pylori* infection
cc3	18-01-1990/F	H-r1	Daughter 1	20.9	*Helicobacter pylori* infection
cc4	27-09-1995/F	H-r2	Daughter 2	22.9	None
cc5	18-11-1962/F	H-u1	Unrelated	19.1	None
cc6	07-07-1978/F	H-u2	Unrelated	23.4	*Helicobacter pylori* infection
cc7	28-12-1971/F	TCD-u	Unrelated	21.7	Seasonal atopy
cc8	01-01-1942/F	UCD-u	Unrelated	18.5	Osteoporosis Colonic diverticulosis

*F, Female; M, Male; H, Healthy Control; TCD, Treated Celiac Disease; UCD, Untreated Celiac Disease; r, related; u, unrelated*.

More in depth, cc1 (the mother) is a treated celiac patient (TCD, “treated celiac disease”). She is also referred to as TCD-related (TCD-r, Table 1) to underline that she belongs to the family. She has been following a strict gluten-free diet since the diagnosis 8 years earlier, thus her CD serology was negative with mucosal recovery at the time of the study. The father (cc2) suffered from untreated celiac disease (UCD) and is referred to as UCD-related (UCD-r). He always refused the GFD, thus both serological and histological markers were persistently positive. The two daughters (cc3 and cc4) are indicated as healthy-related (H-r1 and H-r2). They both had negative serologic results in the absence of class A immunoglobulin deficiency with an increase in intraepithelial lymphocyte counts (respectively 41/100 and 33/100 epithelial cells) at duodenal histology.

Among the four unrelated subjects, cc5 and cc6 are non-celiac controls diagnosed with functional dyspepsia and are also referred to as healthy-unrelated (H-u1 and H-u2, Table 1). cc7 and cc8 are celiac patients, the former under a gluten-free diet since three years and the latter a neo-diagnosed patient. They are thus indicated as TCD-u (cc7) and UCD-u (cc8), respectively.

**Figure 1:**
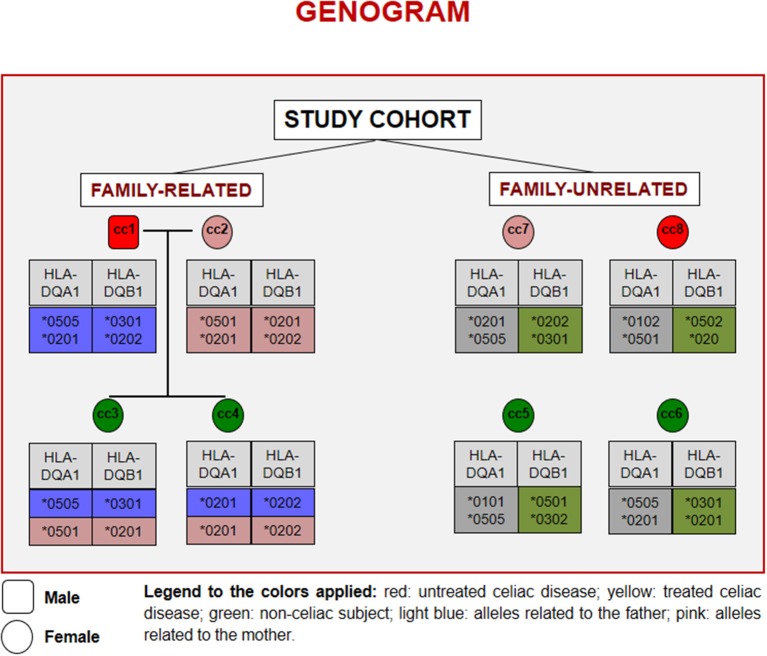
Genogram representing the cohort of subjects recruited for the study. Familiar relations and HLA alleles at the DQA1 and DQB1 loci are reported.

Figure 1 reports the HLA haplotypes of the enrolled subjects. cc1 and cc3 carry two heterodimers of the classical DQ2 haplotype, while cc2 and cc6 present one heterodimer of the DQ2 haplotype, as cc7 and cc8. Both daughters (cc3 and cc4) inherited different combinations of the parental HLA loci. Finally, cc4 carries two alleles of the monomer β of the DQ2 haplotype, while cc5 displayed the monomer α of the DQ2 and a monomer β of the DQ8 susceptibility haplotypes.

### Sample collection

A 10 mL sample of whole peripheral blood was drawn from each subject at the Gastroenterology Unit, Policlinico G.B. Rossi, AOUI Borgo Roma (Verona, Italy) and collected in EDTA for immediate processing. Sample collection was conducted in accordance with the World Medical Association's Declaration of Helsinki, and it was approved by the local Ethics Committee for Clinical Sperimentation (CESC) of Verona and Rovigo. Each enrolled subject gave written informed consent to participate in the study and did not have any acute illness at the time of blood harvest unless those specified in Table 1.

### Peripheral blood mononuclear cells and RNA isolation

PBMCs were purified using the Lympholyte®-H (Cedarlane; Toronto, Canada) density gradient. Total RNA was then extracted with a trizol-based procedure (TRI Reagent, Sigma Aldrich; St. Louis, MO). RNA was quantified using a NanoDrop spectrophotometer (Agilent; Santa Clara, CA) and quality-checked using a Bioanalyser 2100 (Agilent, RNA 6000 Nano Assay).

### Microarray hybridization and data analysis

Microarray experiments were performed by the Genopolis Consortium (University of Milano-Bicocca; Milan, Italy) using the Affymetrix Human U133PLUS2 GeneChip®technology (54K probes) according to standard protocols. Hybridized arrays were scanned using the GeneArray scanner, and the resulting images analyzed with the Affymetrix GeneChip Operating Software (GCOS). Quality scores were then inferred for each spot, background subtracted with the GCRMA algorithm, and signal intensities normalized using qSpline (25). Quality-filtering was performed by removing all probesets whose values were not significantly different from control values or below the 95th percentile. Differentially expressed genes (DEG), i.e., genes whose expression resulted significantly different between a given sample and the one chosen as the reference (common baseline), were selected among the quality-filtered probesets. The FoldChange method was used and expression values expressed as log_2_ ratios. Hierarchical clustering and heatmap analyses were used to group samples by their expression patterns. Finally, the Partitioning Around Medoid-clustering method (26) was used to partition the gene expression profiles into k clusters of genes presenting similar regulation profiles among samples.

### Gene and pathway analysis

To define biological functions and processes associated with DEG, information from ENSEMBL (ensembl.org) and NCBI (ncbi.nlm.nih.gov) was mutated. Within NCBI, the OMIM (Online Mendielian Inheritance in Man) and UniGene datasets were screened. Gene pathways were analyzed according to either the Kyoto Encyclopedia of Genes and Genomes (KEGG, genome.jp/kegg) and the Gene Ontology (GO, http://www.informatics.jax.org) tools. The *p*-value was calculated for the pathways associated with DEG of the various clusters, against the null hypothesis that the enrichment for a particular pathway is random. A *p*-value < 0.05 was considered statistically significant.

## Results

### Microarray analysis

**Figure 2:**
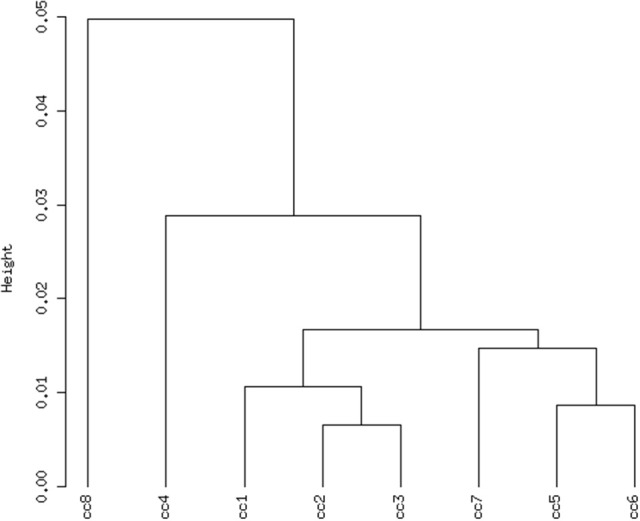
Hierarchical clustering of arrays. The vertical scale indicates Pearson correlation coefficients as a measure of similarity. cc1 is the treated celiac family member (mother); cc2 is the untreated celiac family member (father); cc3 is a healthy family member (elder daughter); cc4 is a healthy family member (youngest daughter); cc5 is the unrelated healthy control used for comparison; cc6 is an unrelated healthy control; cc7 is an adult unrelated treated celiac disease patient; cc8 is an adult unrelated untreated celiac disease patient. For comments, see the text.

Total RNA extracted from PBMCs of the eight subjects was used for the microarray hybridization. A total of 20,300 out of 54,675 probesets on the array passed the filters and underwent downstream analyses. Samples were first grouped on the basis of their overall expression profiles, and a distance measurement was used to verify the quality of replicas. Resulting dendrograms provide an unbiased overview of the relationships among samples on the basis of their overall gene expression profile (Figure 2). It is worth noting that the family members cc1–cc4 clustered together, regardless of their disease status. The sharing of a common household environment and a partially overlapping genetic background seem to shape the overall PBMC gene expression more than the CD status. On the contrary, the clustering of samples belonging to unrelated individuals cc5–cc8 was dictated by the disease status. The UCD-u patient (cc8) resulted the most diverse, while TCD-u (cc7) shared more similarities with the two H-u (cc5 and cc6). No intermix was observed between the family members and the external controls.

### Selection and clustering of DEG

**Figure 3:**
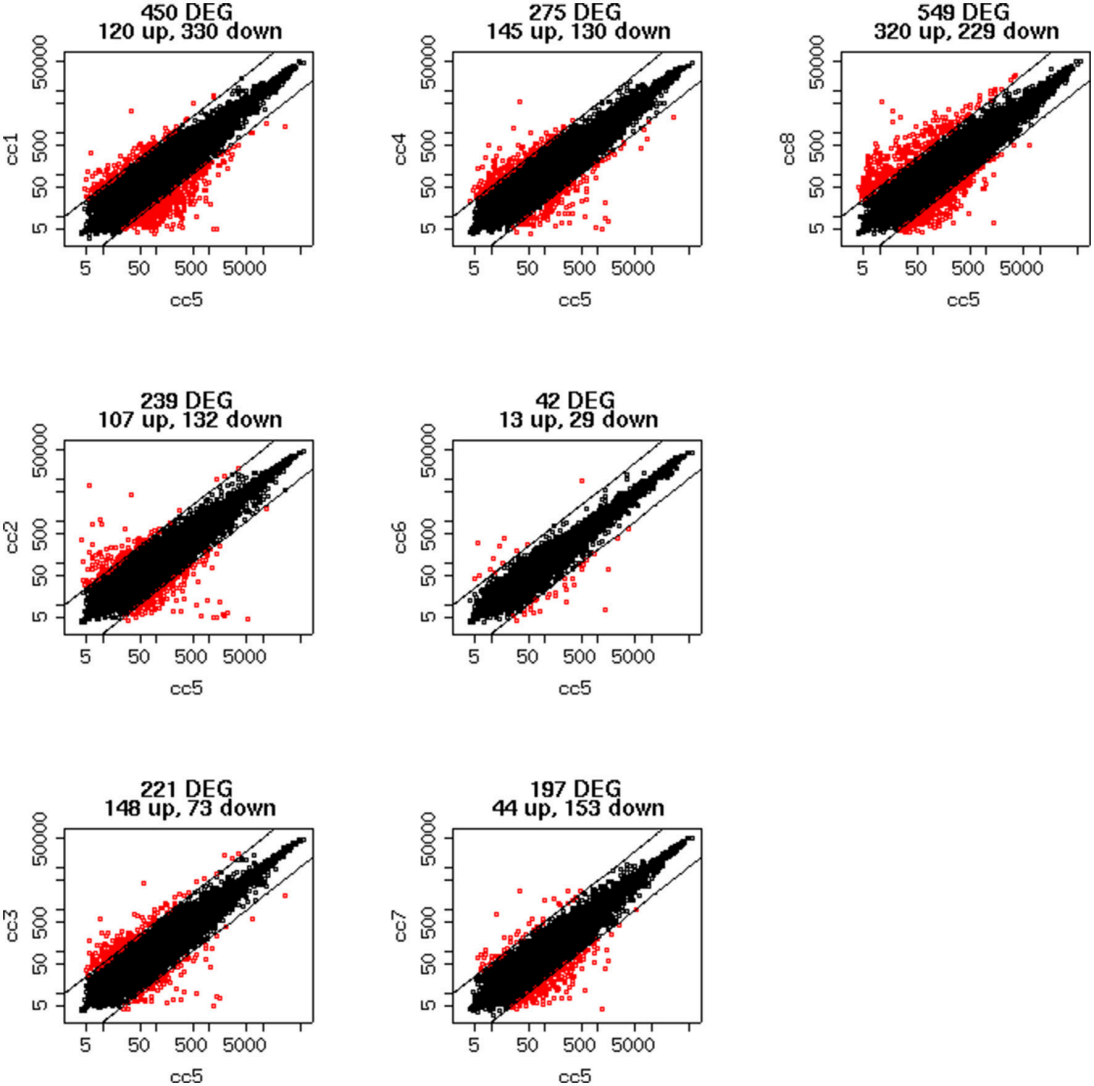
Scatter plots of the differentially expressed genes per subject. The y-axis shows the mean of the replicates for that sample, and the x-axis is the mean across the replicates of the sample chosen as a “reference” (cc5). Red points represent the differentially expressed genes. The distance between the two black lines is the cutoff that defines differently expressed genes (red points).

**Figure 4:**
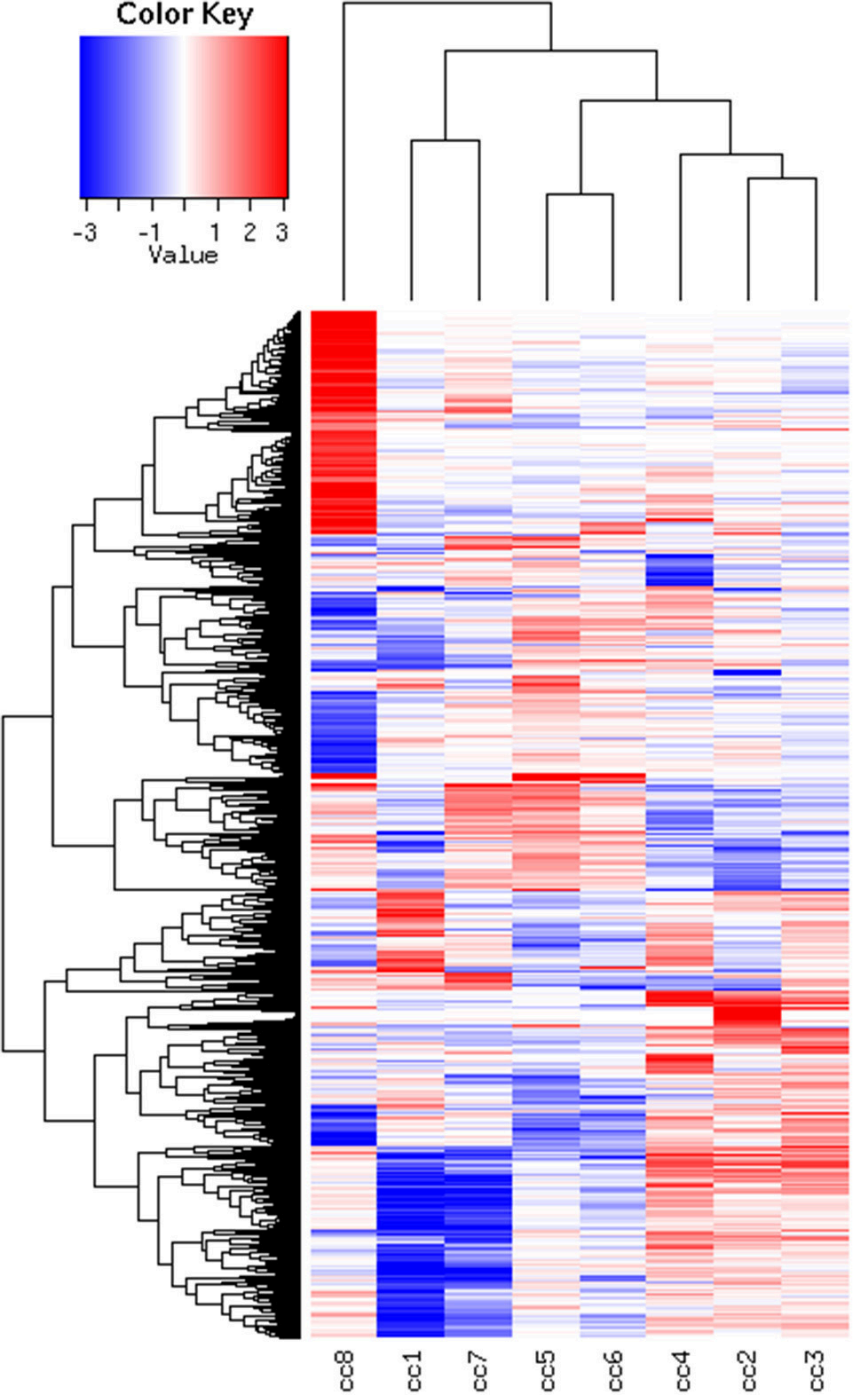
Heatmap of the DEG universe per subject. Each probeset is differentially expressed in at least one condition. The log2 of the ratio between each value and the median of the row is reported on a blue-red scale.

To identify DEG, the FoldChange method was used, with the H-u1 control (cc5) chosen as the baseline (“reference”). Figure 3 presents the scatter plots of the DEG identified in each sample: a total of 1,360 unique probesets resulted differentially expressed, some of them in multiple samples. The number of DEG ranged from a minimum of 42 observed in cc6 (the other control external to the family, resulted the most similar to the reference cc5 in terms of overall gene expression profile) to a maximum of 549. This number was observed in cc8, the UCD-u case. Notably, this was also the patient that clustered alone in the dendrogram shown in Figure 2. A further consideration is due on the fact that cc8 is also the sample with the highest number of up-regulated genes, whereas cc1 (TCD-r) presents the highest amount of down-regulated genes. These data are confirmed by the heatmap analysis shown in Figure 4. From the figure, it clearly emerges that the condition of “treated CD” is associated with a wide “down-regulation picture” in both cc1 and cc7, while the situation appears more heterogeneous for the two active CD cases. Indeed, cc8 (the most diverse sample according to Figure 2) is characterized by a conspicuous group of up-regulated genes while in cc2 the situation is more nuanced and closer to the daughters cc3 and cc4. f

**Figure 5:**
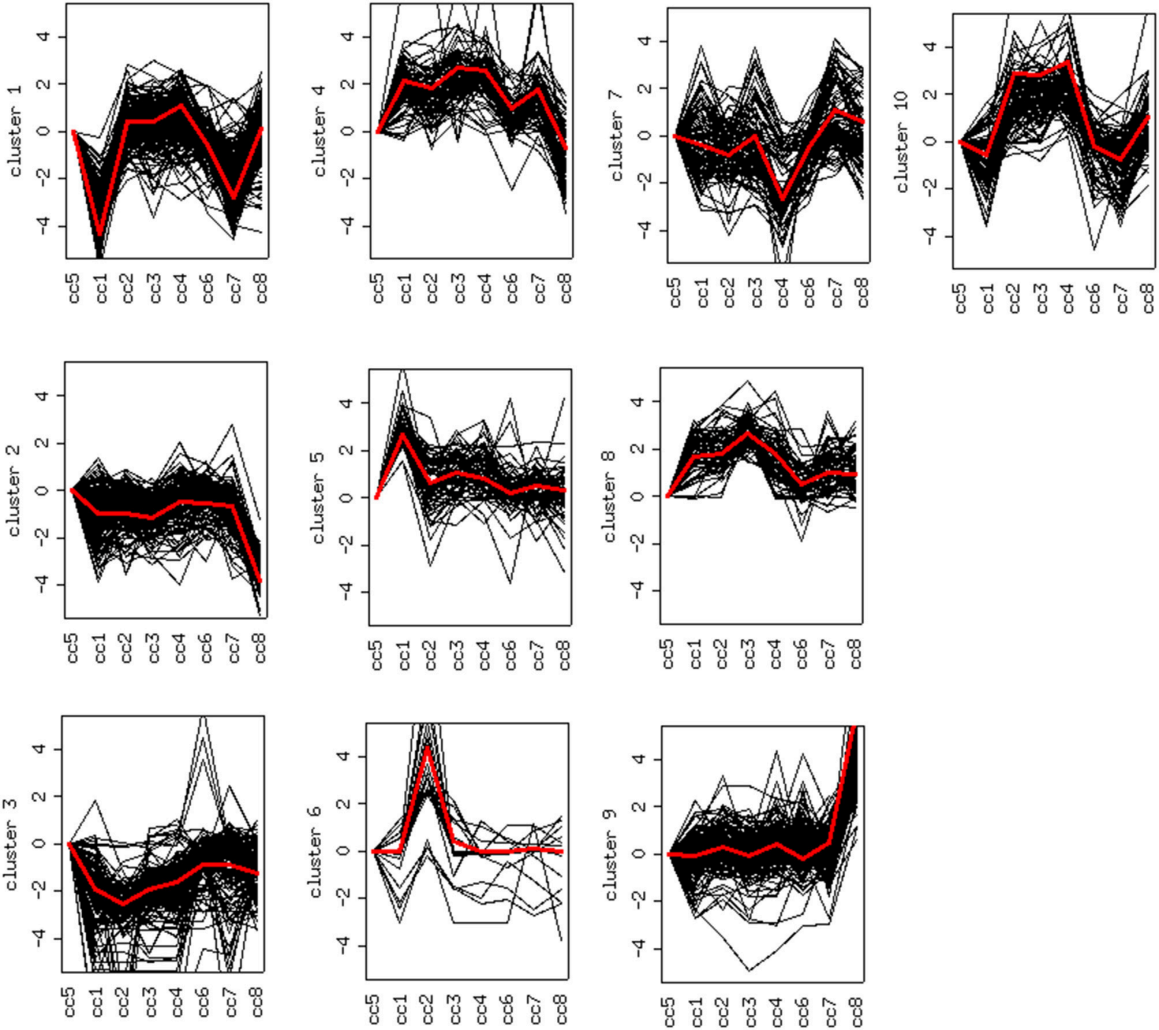
Clustering of differentially expressed genes. Expression profiles of genes belonging to the 10 clusters that partition the dataset (log2 ratios). The red line represents the general pattern that characterizes each cluster. The Y axis represents up- and down-regulations. The X axis reports samples (cc1–cc8). The “baseline” sample (cc5) is the first on the left: the corresponding expression values are always assumed to be = 0.

DEG displaying similar expression trends across the different samples were then grouped into clusters (26). This analysis is based on the assumption that the similarity of gene expression profiles within a given cluster is suggestive of a similar regulation. Based on this approach, 10 clusters were found to optimally partition the dataset. Panels in Figure 5 report the expression trends, along the experimental cohort, of genes belonging to each cluster, expressed as log_2_ ratios vs. the reference cc5. Among the ten clusters, several present interesting behaviors in relation to the cohort of cases. Clusters 1, 2, 6, 9, and 10 are discussed below.

### Cluster 1: off switch in celiac patients on a GFD

Cluster 1 groups strongly down-regulated genes in both TCD cases (cc1 and cc7). Supplementary Table 1 lists the 80 probes whose log_2_ ratio calculated on cc5 was < −4 in both patients. The relative expression with respect to the two UCD cases (cc2 and cc8) was also assessed. A large number of these genes is linked to the regulation of gene expression and to cell proliferation: the resulting picture indicates an overall silencing in TCD. Interestingly, many down-regulated genes were involved in cell-cell and cell-matrix interactions, vesicular and molecular trafficking, exosomes and endosomes routes, protein ubiquitination and turnover. As shown in Supplementary Table 1, in general the situation did not change if calculating expression ratios vs. either cc2 or cc8 instead of cc5. This suggests that in active CD there is a pattern similar to healthy controls and that this pattern is then shut down following the GFD. Table 2 reports the functional annotation of these genes, and specifically the KEGG pathways resulting significantly enriched among genes belonging to cluster 1. This analysis basically confirms the scenario depicted above. From a statistic point of view, the KEGG pathways most significantly enriched in cluster 1 are: the JAK-STAT signaling (*p* = 0.00064) followed, among others, by the T-cell receptor cascade (*p* = 0.018), the Toll-like receptor signaling (*p* = 0.0245) and the focal adhesion (cell-matrix adhesion, *p* = 0.0302).

**Table 2:** Kyoto Encyclopedia of Genes and Genomes (KEGG) annotation of differentially expressed genes (DEG) belonging to cluster 1 (genes down-regulated in treated celiac disease patients: cc1 and cc7).

**KEGG ID**	**Description**	***p***
04630	Jak-STAT signaling	0.00064
04660	T cell receptor pathway	0.018
04640	Hematopoietic cell lineage	0.0238
04620	Toll-Like receptor signaling	0.0245
04510	Focal adhesion (cell-matrix adhesions)	0.0302
04060	Cytokine-receptors interactions	0.0358
04210	Apoptosis	0.0433
04810	Regulation of actin cytoskeleton	0.0445
04910	Insulin signaling	0.0471

*Only KEGG pathways that result significantly regulated (p-value < 0.05 against the null hypothesis that their enrichment in DEG is random) are given in the Table*.

### Clusters 2, 6 and 9: deregulation in active celiac disease

Cluster 2 groups genes down-regulated in cc8, the UCD-u case. Supplementary Table 2 reports the probes for which the log_2_ of the ratio vs. cc5 was < −4. For the same genes, the relative expressions vs. the TCD cases (cc1 and cc7) and vs. one healthy daughter (cc4) are reported. As above, nearly all of the genes result down-regulated at comparable levels in all cases. A number of genes belonging to cluster 2 regulate cell cycle, proliferation and apoptosis. A small group belongs to the MAP kinase (MAPK) family. Other well-represented groups are genes involved in cytoskeleton assembly and functions and protein/vesicular trafficking at all levels (specifically: endosome internalization and intracellular trafficking, transport of proteins to the endoplasmic reticulum, post-Golgi transport, protein docking and exocytosis, recognition of misfolded proteins and ubiquitin cycle, cytoskeleton assembly and tight junctions). Finally, other genes are implied in energy metabolism or in phosphatidylinositol signaling. Table 3 presents the KEGG functional annotation of these genes. The strongest result reported in this table concerns the energy metabolism, and namely, the citric acid cycle (*p* = 8.85 × 10^−7^). The MAPK signaling pathway (*p* = 0.0145) is also worth of mentioning. Concerning the protein and vesicular trafficking, further details were obtained through the GO annotation. Among others, the following resulted significantly enriched among DEG belonging to cluster 2: Golgi vesicle transport (GO:0048193, *p* = 2.38 × 10^−8^); intracellular transport (GO: 0046907, *p* = 8.08 × 10^−8^) and intracellular protein transport (GO:0006886, *p* = 2.26 × 10^−5^); secretory pathway (GO:0045045, *p* = 9.62 × 10^−7^); vesicle-mediated transport (GO:0016192, *p* = 1.36 × 10^−5^); endoplasmic reticulum to Golgi transport (GO:0006888, *p* = 2.61 × 10^−4^).

**Table 3:** Kyoto Encyclopedia of Genes and Genomes (KEGG) annotation of differentially expressed genes (DEG) belonging to cluster 2 (genes down-regulated in the untreated celiac disease patient: CC8).

**KEGG ID**	**Description**	***p***
00020	Citrate cycle (TCA cycle)	8.85 × 10^−7^
04620	Toll-Like receptor signaling	0.000143
00760	Nicotinate and nicotinamide metabolism	0.00213
00564	Glycerophospholipid metabolism	0.00488
04010	MAPK signaling pathway	0.0145
04660	T cell receptor signaling pathway	0.0163
04514	Cell adhesion molecules (CAMs)	0.0385

*Only KEGG pathways that result significantly regulated (p-value < 0.05 against the null hypothesis that their enrichment in DEG is random) are given in the Table*.

Cluster 6 includes a small number of genes with a very strong up-regulation in cc2 (Supplementary Table 3). Unlike the previous clusters, these genes do not share a function: accordingly, no KEGG pathways resulted significantly overrepresented. Instead they appeared to share a common cytogenetic location on the long arm of chromosome Y, in the region known as MSY (male-specific region of the Y chromosome) (27). This region comprehends genes expressed in various tissues, including also peripheral leukocytes (28).

Finally, cluster 9 includes a large group of genes up-regulated in UCD-u (cc8, Supplementary Table 4). In this case, cell proliferation appeared to be strongly increased (see for example the up-regulation of histone genes and factors promoting proliferation and differentiation of hematopoietic precursors). Another remarkable series of up-regulated genes are linked to inflammation (cell adhesion molecules, prostaglandins, leukotrienes, chemokines, and chemotaxis). A further well-represented class represents genes encoding coagulation factors, including the subunits of the heterodimeric von Willebrandt factor. Finally, severe alterations in cytoskeleton, actin-mediated processes, actin/myosin-mediated contraction, protein and vesicular trafficking, protein turnover, focal adhesion, tight junctions and cell-matrix interactions emerged. When computing the expression ratio of these genes with respect to cc1 (TCD-r), it appears that the functions up-regulated in active CD are then silenced under a GFD (Supplementary Table 4). Notably, only a part of these genes were up-regulated also in the other untreated celiac patient, cc2. Indeed, the two UCD of the cohort appeared to have distinct gene expression pictures, as also evident from the clustering results in Figure 2 and the heatmap in Figure 4. Table 4 presents the KEGG pathways significantly enriched among DEG belonging to this cluster. These present particularly significant *p*-values, as in the case of prostaglandin and leukotriene metabolism (inflammation, *p* = 8.19 × 10^−6^); extra-cellular matrix-receptor interaction (*p* = 8.06 × 10^−10^); cell differentiation along the haematopoietic lineage (*p* = 8.37 × 10^−9^) and focal adhesion (*p* = 3.35 × 10^−5^). Concerning, the coagulation processes, the analysis conducted using the GO database provided highly significant results: among others, GO:0050817 (coagulation) and GO:0030168 (platelet activation) resulted enriched with *p*-values respectively of 7.72 × 10^−11^ and = 1.98 × 10^−5^).

**Table 4:** Kyoto Encyclopedia of Genes and Genomes (KEGG) annotation of differentially expressed genes (DEG) belonging to cluster 9 (genes up-regulated in the untreated celiac disease patient: CC8).

**KEGG ID**	**Description**	***p***
04512	Extra-cellular matrix-receptor interaction	8.06 × 10^−10^
04640	Hematopoietic cell lineage	8.37 × 10^−9^
00643	Styrene degradation	8.74 × 10^−7^
00590	Prostaglandin and leukotriene metabolism	8.19 × 10^−6^
04510	Focal adhesion	3.35 × 10^−5^
04610	Complement and coagulation cascade	0.000131
04810	Regulation of actin cytoskeleton	0.000529
04330	Notch signaling	0.00128
04540	Gap junction	0.00951
00350	Tyrosine metabolism	0.011
00230	Purine metabolism	0.019
04020	Calcium signaling pathway	0.0193
04060	Cytokine-cytokine receptor interaction	0.0262
00564	Glycerophospholipid metabolism	0.0267

*Only KEGG pathways that result significantly regulated (p-value < 0.05 against the null hypothesis that their enrichment in DEG is random) are given in the Table*.

### Cluster 10: a family effect or first signs of future disease?

Cluster 10 is a complex “familial” group of genes, with the parents exhibiting opposite behaviors (cc1 down-regulated and cc2 strongly up-regulated) and the daughters cc3-cc4 also displaying an up-regulation, but to a minor extent. Supplementary Table 5 lists the genes whose log_2_ was >4 for the ratio cc2/cc5 and/or < −4 for the ratio cc1/cc5. The major contribution to this list comes from genes strongly up-regulated in cc2, and generally slightly down-regulated in cc1. These genes fall into a few, already mentioned, categories: cell cycle regulators (apoptosis, DNA methylation, chromatin remodeling), gene expression (transcription factors and mostly post-transcriptional regulation of mRNA stability), actin cytoskeleton, vesicular and protein trafficking. The analysis of genes up-regulated in cc2 broadly reproduces the picture of activation of cluster 9, particularly regarding cell proliferation. On the other hand, the same genes were down-regulated in both TCD patients in cluster 1. Table 5 reports the KEGG pathways that were significantly enriched in this cluster.

**Table 5:** Kyoto Encyclopedia of Genes and Genomes (KEGG) annotation of differentially expressed genes (DEG) belonging to cluster 10 (genes up-regulated in c2-c4 and down-regulated in c1).

**KEGG ID**	**Description**	***p***
04110	Cell cycle	0.00181
04510	Focal adhesion	0.00432
04810	Regulation of actin cytoskeleton	0.00472

*Only KEGG pathways that result significantly regulated (p-value < 0.05 against the null hypothesis that their enrichment in DEG is random) are given in the Table*.

**Figure 6:**
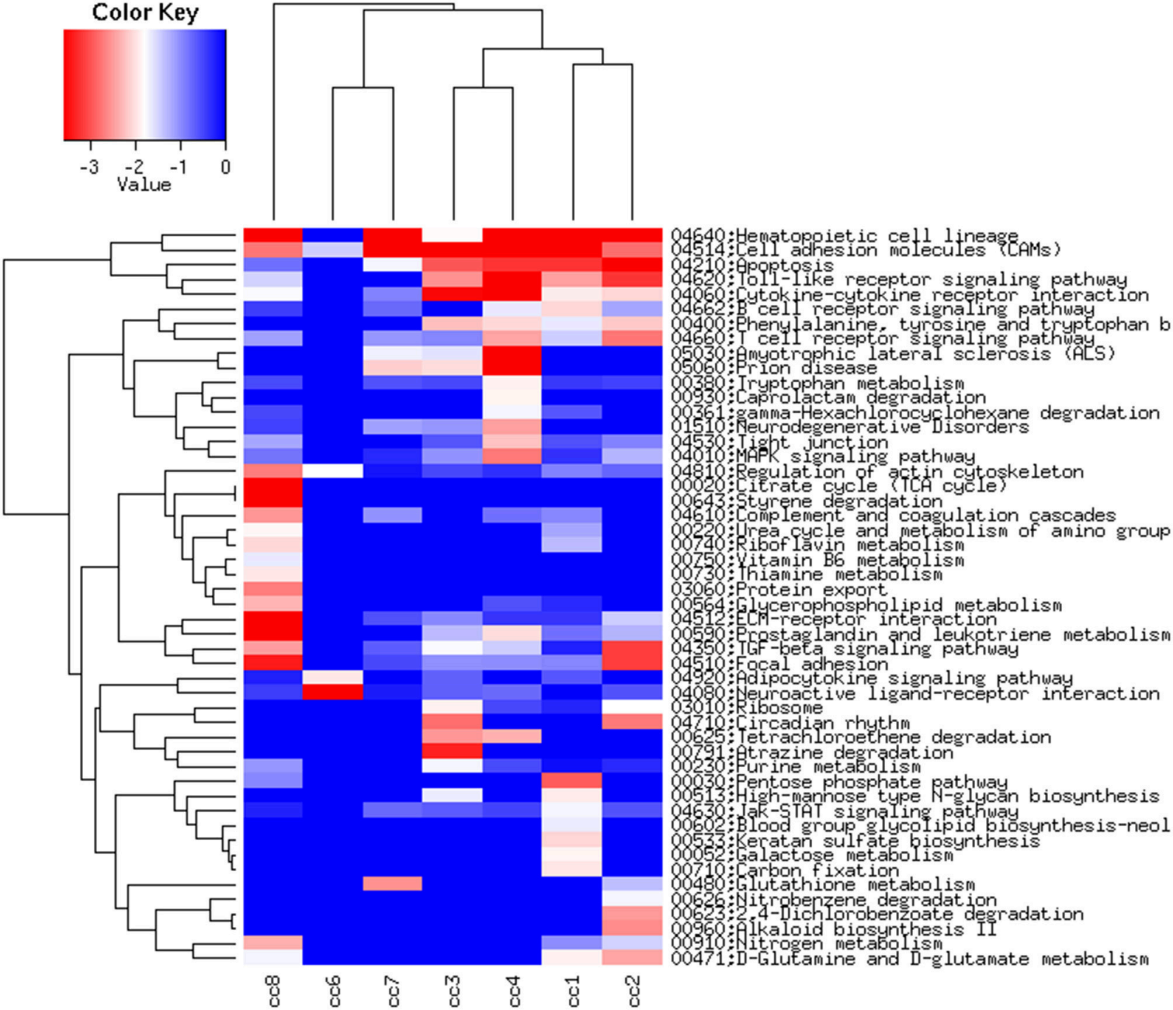
Heatmap of the KEGG pathways per subject. The log2 of the ratio between each value and the median of the row is reported on a blue-red scale.

Figure 6, presenting the heatmap analysis performed on the KEGG pathways, summarizes all the findings described above.

## Discussion

The unique possibility to study the naturally-occurring experimental model of a family where both parents were suffering from CD (one active and one treated) while their two daughters were not, has allowed us to address the PBMC gene expression in celiac patients in an original way. Within this family, factors other than the classical genetic susceptibility may have contributed to the development of an overt disease in two out of four members. Four unrelated individuals were added to the study, all of which carried the same genetic susceptibilities to celiac disease found within the family. These non-related individuals were meant to better unravel the relevance of factors other than genetics and comprehended two controls and two celiac patients (one under gluten-free diet) to mirror what was occurring within the family. To our knowledge, a comparable cohort of patients has never been addressed and analyzed to define the PBMC gene expression, and this represent a novelty that this study brings to the literature, despite its limitations. These are linked to the fact that a limited number of subjects is included and that the work reflects a unique, non-repeatable situation that ended in the past. Indeed, one member of the family, namely the father, died from a heart attack a few months later being enrolled in this study, the mother developed a B-cell lymphoma localized in the central nervous system, while the clinical findings of the others (especially the daughters) meanwhile have probably evolved and are no longer similar to what we have pictured through the microarray analysis presented here.

The overall picture emerging from the analysis of PBMC gene expression on one hand confirms and enlarges several previous findings, referring either to intestinal mucosa and blood, and on the other hand provides useful hints to pathways/mechanisms implied in CD pathogenesis.

The PBMC gene expression picture that emerged in TCD, in accordance with published data (29) mirrors an overall silencing of several functions, including cell-matrix interactions, vesicular and molecular trafficking, cell activation and proliferation (clusters 1 and 10). This could be suggestive of the fact that the GFD does not restore PBMCs to a “physiological” status (30), but rather it switches off abnormally activated pathways.

The situation in UCD appears specular: cluster 9 and, to a minor extent, cluster 10 show how cell proliferation is strongly pushed (31), presumably in compensation for the exaggerated apoptosis affecting the intestinal mucosa (32). Looking in particular at Cluster 10, it seems reasonable to hypothesize that both daughters (cc3 and cc4) are at high risk of developing a gluten-dependent enteropathy, thus they are followed closely with serological and clinical monitoring every six months. At present they still have a negative serology.

Two notable groups of genes up-regulated in UCD (cluster 9) are linked to inflammation (i.e., cell adhesion molecules, prostaglandins, leukotrienes, chemokines, and chemotaxis) and blood coagulation. This is consistent with data on other inflammatory conditions (33). For example, in dermatitis herpetiformis, the so-called CD of the skin (1), the contemporary activation of these two pathways is thought to represent a pathogenic mechanism (34). Additionally, impaired coagulation in UCD has recently been reported. This leads to a hypercoagulability status and increased venous thromboembolism, with an augmented stroke risk (35). Finally, one of the genes involved in the inflammatory process (OLFM4) is already reported to be up-regulated in PBMCs together with inflamed colonic mucosa in active ulcerative colitis (30).

Severe alterations were also found in cytoskeleton and its functions, focal adhesion, tight junctions and cell-matrix interactions, actin-mediated processes and actin/myosin contraction, protein and vesicular trafficking at all levels. This is consistent with several recent reports (29, 36–39).

In particular, the differential expression of contraction genes (in addition to cell proliferation and tight-junction genes) has been reported in the intestinal mucosa and PBMCs of pediatric CD cases (32), with some genes being down-regulated and others up-regulated, and normalization after two years of GFD. In addition, the expression of tight junction proteins resulted dysregulated in biopsies from UCD (38, 39). Finally vesicular trafficking has been found to be heavily impaired in CD, mainly as a consequence of the binding of actin filaments to gliadin (29, 40).

The overall picture of strong impairment of cytoskeleton structures and protein/vesicular trafficking in PBMC of active celiac patients is indicative of a passage of “information” from the inflamed intestinal mucosa to peripheral blood cells. In other words, our data depict a situation where PBMCs of UCD patients are experiencing an intense cross-talk with the gut mucosa, that is then silenced after the introduction of a GFD. This cross-talk is possibly mediated by exosomes.

Linked to this, the finding on the up-regulation, in UCD, of genes coding for the heterogeneous ribonuclear protein family (cluster 10). These genes have been precisely proposed to control sorting and inclusion of miRNA into exosomes (41). The remarkable number of over-expressed genes linked to protein ubiquitination is probably also linked to this. Indeed, it is known that sorting into exosomes of integral membrane proteins occurs through ubiquitination (19). Among the membrane integral proteins known to be included into exosomes, it is worth mentioning the HLA class II proteins, included the cases where they are bound to food antigens (22, 23).

Our data also witness the strong dysregulation of MAPK signaling and energy metabolism in CD. Energy metabolism, in particular the citric acid cycle, glycolysis and amino acid metabolism, resulted impaired in a recent NMR-based study of the gastrointestinal mucosa of active CD (42). Western-blot analysis on PBMCs of UCD patients confirmed that MAPK signaling was down-regulated (43).

In summary our results depict an overall switching-off in TCD and hyper-activation in UCD, where dangerous signals are likely spread from the inflamed intestinal mucosa (22) to other sites in the body by circulating blood cells (16, 17).

Finally, it is worth mentioning the finding on the strong up-regulation, in cc2, of physically adjacent genes located in the same region of the Y chromosome, the so-called MSY. This region is reportedly associated with coronary artery disease (44), and the up-regulation of specific genes within it (namely EIF1AY and USP9Y, as found in cc2, see Supplementary Table 3) specifically linked with idiopathic heart failure (45). Indeed, cc2 died suddenly from myocardial infarction. We think that these preliminary findings could deserve further investigation as adulthood CD is reportedly associated with an increased cardiovascular morbidity, whose pathogenic bases are still poorly understood (46, 47).

In conclusion, our results highlight an extensive, gluten-dependent, dysregulation, in peripheral blood cells, in the expression of genes encoding for a number of pathways that contribute to CD pathogenesis, together with a strong effect of the environment in shaping their expression. Furthermore, microarray technology still appears a powerful tool for investigating new therapeutic targets, and circulating mononuclear cells were confirmed to be an essential source of information on mucosal immunity in celiac patients.

## Datasets are available on request

The raw data supporting the conclusions of this manuscript will be made available by the authors, without undue reservation, to any qualified researcher.

## Author contributions

RC had responsibility of the diagnostic and therapeutic management of the patients; RC and SP contributed conception and design of the study, wrote the paper and critically revised the literature; MC and GC collected clinical samples and data; EC and SP analyzed and interpreted the data; GC critically revised the manuscript for important intellectual contents. All authors contributed to manuscript revision, read and provided approval for publication of the submitted version and agreed to be accountable for all aspects of the work in ensuring that questions related to the accuracy or integrity of any part of the work were appropriately investigated and resolved.

### Conflict of interest statement

The authors declare that the research was conducted in the absence of any commercial or financial relationships that could be construed as a potential conflict of interest. The reviewer GL and handling Editor declared their shared affiliation.

## Abbreviations

AURE: AU-rich elements
CD: Celiac disease
DEG: Differentially expressed genes
GFD: gluten-free diet
H-r: healthy-related
H-u: healthy-unrelated
HLA, Human Leukocyte Antigen: : KEGG: Kyoto Encyclopedia of Genes and Genomes
MAPK: MAP kinase
MSY: male-specific region of the Y chromosome
PBMC: peripheral blood mononuclear cells
TCD: treated celiac disease
TCD-r: treated celiac disease-related
TCD-u: treated celiac disease-unrelated:
UCD: untreated celiac disease
UCD-r: untreated celiac disease-related
UCD-u: untreated celiac disease-unrelated.
