# Supplementary material for: The Transcriptomic Analysis of Circulating Immune Cells in a Celiac Family Unveils Further Insights Into Disease Pathogenesis
Source: Front Med (Lausanne). 2018 Jun 19;5:182. doi: 10.3389/fmed.2018.00182 (PMC6018082; doi:10.3389/fmed.2018.00182)
Supplement: Supplementary file 1 [file Data_Sheet_1.DOCX]

Supplementary Material

**THE TRANSCRIPTOMIC ANALYSIS OF CIRCULATING IMMUNE CELLS IN A CELIAC FAMILY UNVEILS FURTHER INSIGHTS INTO DISEASE PATHOGENESIS**

*^§1^Rachele Ciccocioppo, ^§2^Simona Panelli, ^1^Maria Cristina Conti Bellocchi, ^3^Giuseppina Cristina Cangemi, ^1^Luca Frulloni, ^4^Enrica Capelli, ^3^Gino Roberto Corazza

^§^These authors contributed equally to the work

^1^Gastroenterology Unit, Department of Medicine, AOUI Borgo Roma, University of Verona, Verona, Italy; ^2^Pediatric Clinical Research Center “Invernizzi”, University of Milano, Milano, Italy; ^3^Clinica Medica I, Department of Internal Medicine, Fondazione IRCCS Policlinico San Matteo, University of Pavia, Pavia, Italy; ^4^Department of Earth and Environmental Sciences, University of Pavia, Pavia, Italy.

**Correspondence:** Prof. Rachele Ciccocioppo M.D., Gastroenterology Unit, Department of Medicine, AOUI Borgo Roma, University of Verona; Piazzale L.A. Scuro, 10, 37134 Verona, Italy. Tel. +39 (0)45 8124578. Fax +39 (0)45 8027495.

Electronic address: [rachele.ciccocioppo@univr.it](mailto:rachele.ciccocioppo@univr.it)

**Supplementary Table 1. Annotated differentially expressed genes (DEG) belonging to cluster 1 (genes down-regulated in treated celiac disease patients: cc1 and cc7).** Only genes that present a log_2_ ratio (calculated using cc5 as reference) <-4 are shown in the table. Relative expressions with the two untreated celiac disease patients (cc2 and cc8) are also shown, always as log_2_ values. Details for each gene are given on the basis of their Gene Ontology annotation.

| **Symbol** | **Description** | **GenBank** | **Cytoband** | **Details** | **C1/C5** | **C7/C5** | **C1/C2** | **C7/C2** | **C1/C8** | **C7/C8** |
| --- | --- | --- | --- | --- | --- | --- | --- | --- | --- | --- |
| ***Cell cycle regulators, transcription factors, regulators of gene expression*** | | | | | | | | | | |
| JAK2 | Janus kinase 2 | BC043187 | 9p24 | regulation of cycle; JAK-STAT cascade | -4,88 | -4,03 | -4,81 | -3,96 | -5,21 | -4,36 |
| SPEN | Homolog of *Drosophila* spen | AL524033 | 1p36.33-p36.11 | Transcription regulation through the Notch pathway | -5,84 | -6,15 | -6,10 | -6,41 | -6,31 | -6,62 |
| CDK6 | Cyclin-dependent kinase 6 | AW274756 | 7q21-q22 | Regulation of cell cycle (G1 phase) | -5,49 | -4,49 | -5,88 | -4,88 | -5,62 | -4,62 |
| NCOA3 | Nuclear receptor coactivator 3 | U80737 | 20q12 | Transcription factor | -5,48 | -4,08 | -5,57 | -4,18 | -4,89 | -3,49 |
| JARID1A | Jumonji, AT rich interactive domain 1A | AF007135 | 12p11 | Transcription factor | -5,85 | -4,71 | -5,48 | -4,33 | -5,21 | -4,06 |
| MLL3 | Myeloid/lymphoid or mixed-lineage leukemia 3 | AW137099 | 7q34-q36 | Transcription regulation; chromatin modification | -6,79 | -5,41 | -7,35 | -5,97 | -7,37 | -5,99 |
| MALAT1 | Metastasis associated lung adenocarcinoma transcript 1 (non-coding RNA) | AW005982 | 11q13.1 | Post-transcriptional regulation | -5,70 | -4,94 | -6,16 | -5,40 | -5,33 | -4,56 |
| PPARBP | PPAR binding protein | AI423072 | 17q12-q21.1 | Ligand-dependent nuclear receptor: transcriptional activation; responsive to various hormones | -5,81 | -4,44 | -5,96 | -4,60 | -5,73 | -4,37 |
| UHMK1 | U2AF homology motif (UHM) kinase 1 | AW173222 | 1q23.3 | Regulation of cell cycle (arrest);  ubiquitin-mediated proteolysis | -6,27 | -4,81 | -6,50 | -5,05 | -6,70 | -5,25 |
| TULP4 | Tubby like protein 4 | H15278 | 6q25-q26 | Transcription factor: responsive to nutrients | -5,35 | -4,86 | -5,44 | -4,95 | -5,84 | -5,35 |
| FOSL2 | FOS-like antigen 2 | AA019641 | 2p23.3 | Transcription factor: regulation of cell death | -5,61 | -6,30 | -5,61 | -6,30 | -5,25 | -5,94 |
| ANKRD28 | Ankyrin repeat domain 28 | N32051 | 3p25.1 | Transcription regulation: interaction with BRCA1 | -6,35 | -4,68 | -6,51 | -4,84 | -7,11 | -5,44 |
| BIRC4 | Baculoviral IAP repeat-containing 4 | BE380045 | Xq25 | Regulation of apoptosis | -4,83 | -4,17 | -4,39 | -3,73 | -0,43 | 0,23 |
| DDX17 | DEAD (Asp-Glu-Ala-Asp) box polypeptide 17 | AW188131 | 22q13.1 | RNA and miRNA processing | -4,81 | -5,05 | -5,26 | -5,50 | -1,27 | -1,51 |
| NARG1 | NMDA receptor regulated 1 | NM_025085 | 4q31.1 | Transcription factor | -5,62 | -5,17 | -5,39 | -4,94 | -2,10 | -1,65 |
| ZNF264 | Zinc finger protein 264 | NM_003417 | 19q13.4 | Transcription factor | -5,96 | -4,59 | -1,68 | -0,30 | -0,75 | 0,62 |
| STK17B | SER/THR kinase 17b | AI221707 | 2q32.3 | Induction of apoptosis | -5,44 | -5,52 | -1,90 | -1,98 | -0,07 | -0,15 |
| MDM4 | Mdm4, transformed 3T3 cell double minute 4, p53 binding protein (mouse) | W93501 | 1q32 | Negative regulation of transcription; negative regulation of cell proliferation (G0 to G1) | -4,50 | -4,81 | -1,87 | -2,18 | -0,34 | -0,66 |
| ***Immune function and coagulation cascade*** | | | | | | | | | | |
| CD44 | CD44 antigen (homing function and Indian blood group system) | W96225 | 11p13 | Cell-matrix and cell-cell adhesion; collagen and hyaluronic acid binding; hematopoietic cell lineage | -4,92 | -5,12 | -5,90 | -6,11 | -1,84 | -2,05 |
| SPN | Sialophorin (CD43) | BC035510 | 16p11.2 | Chemotaxis; negative regulation of adhesion; cell adhesion molecules (CAM) | -6,67 | -5,92 | -6,18 | -5,44 | -0,52 | 0,22 |
| CD69 | CD69 antigen (p60, early T-cell activation antigen) | BF439675 | 12p13-p12 | Cell surface receptor; sugar binding; signal transduction | -4,74 | -5,83 | -4,97 | -6,05 | -4,66 | -5,75 |
| F5 | Coagulation factor V (proaccelerin) | AA910306 | 1q23 | Cell adhesion; blood coagulation | -5,16 | -4,88 | -5,00 | -4,72 | -5,20 | -4,92 |
| ***Signal transduction and transport*** | | | | | | | | | | |
| ALS2CR3 | Juvenile lateral amyotrophic sclerosis candidate | AV705253 | 2q33 | Neurotransmitter binding | -4,80 | -4,59 | -4,96 | -4,75 | -4,70 | -4,49 |
| SLC26A2 | Solute carrier family 26 (sulfate transporter), member 2 | AI025519 | 5q31-q34 | Integral to plasma membrane: sulphate transport | -7,16 | -5,11 | -6,66 | -4,61 | -6,77 | -4,73 |
| PRKCI | Protein kinase C iota | L18964 | 3q26.3 | Intracellular signalling: atypical protein kinase C activity; secretion; vescicle-mediated transport | -5,09 | -4,61 | -5,39 | -4,91 | -5,00 | -4,52 |
| PIK3R1 | Phosphoinositide-3-kinase, regulatory subunit | AI934473 | 5q13.1 | Intacellular signalling: phosphatidylinositol binding | -5,12 | -4,79 | -5,56 | -5,23 | -5,37 | -5,04 |
| AKAP13 | A kinase (PRKA) anchor protein 13 | AI674926 | 15q24-q25 | Membrane-associated signal transduction | -5,16 | -4,45 | -5,33 | -4,62 | -5,45 | -4,74 |
| SLC16A7 | Solute carrier family 16 (monocarboxylic acid transporters), member 7 | NM_004731 | 12q13 | Integral to plasma membrane: pyruvate transport | -5,56 | -5,27 | -6,01 | -5,72 | -1,77 | -1,48 |
| PKN2 | Protein kinase N2 | AI633689 | 1p22.2 | SER/THR kinase | -5,17 | -4,34 | -5,55 | -4,72 | -5,61 | -4,78 |
| ***Cytoskeleton; cell-cell and cell-matrix interactions; molecular trafficking; protein folding and ubiquitination*** | | | | | | | | | | |
| SSH1 | Slingshot homolog 1 (*Drosophila*) | AI651213 | 12q24.11 | Regulation of actin cytoskeleton | -5,92 | -4,58 | -6,57 | -5,24 | -6,26 | -4,93 |
| LYST | Lysosomal trafficking regulator | U84744 | 1q42.1-q42.2 | Endosome to lysosome transport: cellular defense | -6,29 | -6,11 | -6,25 | -6,06 | -7,06 | -6,88 |
| MKLN1 | Muskelin 1 | AK023427 | 7q32 | Cell-matrix adhesion; signal transduction | -5,57 | -5,72 | -6,38 | -6,52 | -5,99 | -6,13 |
| NUPL1 | Nucleoporin like 1 | AA769986 | 13q12.13 | Molecular trafficking in the nuclear membrane | -6,05 | -5,27 | -6,40 | -5,63 | -5,21 | -4,43 |
| ACBD3 | Acyl-Coenzyme A binding domain containing 3 | AI636775 | 1q42.12 | Cytosol Golgi network-associated protein | -4,98 | -4,89 | -0,64 | -0,56 | -0,85 | -0,77 |
| TPR | Translocated promoter region of activated MET oncogene | AW235355 | 1q25 | Nuclear pore; transport; protein-nucleus import | -5,06 | -5,11 | -5,36 | -5,42 | -0,28 | -0,34 |
| ZNF364 | Zinc finger protein 364 | AL530462 | 1q21.1 | Protein ubiquitination | -5,55 | -4,88 | -4,62 | -3,96 | -4,90 | -4,24 |
| SENP6 | SUMO1/sentrin specific peptidase 6 | AA026388 | 6q13-q14.3 | Protein ubiquitination | -5,54 | -4,29 | -6,32 | -5,07 | -6,04 | -4,79 |
| ***Others*** | | | | | | | | | | |
| COX15 | COX15 homolog, cytochrome c oxidase assembly protein (yeast) | AF026850 | 10q24 | Energy metabolism: mitochondrial respiratory chain | -5,32 | -4,24 | -5,42 | -4,34 | -5,63 | -4,55 |
| CEP1 | Centrosomal protein 1 | AA642477 | 9q33-q34 | Structural to centrosome | -4,91 | -4,08 | -4,99 | -4,15 | -5,46 | -4,62 |

**Supplementary Table 2. Annotated differentially expressed genes (DEG) belonging to cluster 2 (genes down-regulated in cc8, the untreated celiac disease patient external to the family).** Only genes that present a log_2_ ratio (calculated using cc5 as reference) <-4 are shown in the table. Relative expressions with cc4 (Hrelated), cc1 and cc7 (treated celiac patients) are also shown, always as log_2_ values. Details for each gene are given on the basis of their Gene Ontology annotation.

| **Symbol** | **Description** | **GenBank** | **Cytoband** | **Details** | **C8/C5** | **C8/C4** | **C8/C7** | **C8/C1** |
| --- | --- | --- | --- | --- | --- | --- | --- | --- |
| ***Cell cycle regulators, transcription factors, regulators of gene expression*** | | | | | | | | |
| PAX8 | Paired box gene 8 | AY007128 | 2q12-q14 | Transcription factor. Nucleotide biosynthesis | -5,23 | -4,78 | -4,79 | -4,82 |
| MBNL1 | Muscleblind-like (*Drosophila*) | AF401998 | 3q25 | RNA binding; splicing regulation | -5,58 | -6,00 | -4,94 | -4,57 |
| CCNL1 | Cyclin L1 | AY034790 | 3q25.31 | Cell cycle regulation | -5,34 | -4,70 | -5,44 | -5,16 |
| DDX42 | DEAD (Asp-Glu-Ala-Asp) box polypeptide 42 | AF147429 | 17q23.3 | RNA and miRNA processing | -5,85 | -5,51 | -5,38 | -5,18 |
| DDX3X | DEAD (Asp-Glu-Ala-Asp) box polypeptide 3, X-linked | AF061337 | Xp11.3-p11.23 | RNA and miRNA processing | -5,07 | -4,79 | -4,90 | -4,60 |
| DHX9 | DEAH (Asp-Glu-Ala-His) box polypeptide 9 | BF313832 | 1q25 | Double-stranded RNA and DNA binding; RNase activity; transcriptional regulation; DNA repair; maintenance of genome stability | -6,14 | -5,70 | -5,12 | -5,07 |
| MCL1 | Myeloid cell leukemia sequence 1 (BCL2-related) | BF594446 | 1q21 | Regulation of apoptosis | -5,24 | -4,87 | -4,89 | -4,29 |
| PDAP1 | PDGFA associated protein 1 | NM_014891 | 7q22.1 | Signal transduction; cell proliferation | -5,08 | -5,18 | -4,72 | -4,86 |
| CBFB | Core-binding factor, beta subunit | AF294326 | 16q22.1 | Transcription factor | -6,20 | -6,40 | -5,86 | -6,00 |
| GADD45B | Growth arrest and DNA-damage-inducible, beta | AF078077 | 19p13.3 | MAPK signalling pathway; cell cycle | -5,30 | -5,05 | -5,09 | -5,67 |
| MAP3K1 | Mitogen-activated protein kinase kinase kinase 1 | AA361361 | 5q11.2 | MAPK signalling; regulation of cell cycle | -4,97 | -4,92 | -4,88 | -4,68 |
| DUSP1 | Dual specificity phosphatase 1 | AA530892 | 5q34 | MAPK signalling; regulation of cell cycle | -5,66 | -4,05 | -4,88 | -4,51 |
| SP1 | Sp1 transcription factor | NM_138473 | 12q13.1 | Regulation of transcription; TGF-beta signalling pathway | -5,62 | -5,39 | -5,11 | -4,89 |
| GTF2I | general transcription factor II | BC004472 | 7q11.23 | Basal transcription factors | -5,93 | -5,95 | -5,57 | -5,48 |
| POLDIP3 | polymerase (DNA-directed), delta interacting protein 3 | Z93241 | 22q13.2 | DNA replication | -4,97 | -4,39 | -4,41 | -4,29 |
| STAT1 | Signal transducer and activator of transcription | M97935_5 | 2q32.2 | TLR and Jak-STAT signalling pathways | -5,25 | -4,45 | -5,40 | -4,71 |
| NCOA2 | Nuclear receptor coactivator 2 | NM_006540 | 8q13.3 | Regulation of transcription | -5,19 | -1,17 | -1,90 | -0,98 |
| CARD12 | Caspase recruitment domain family, member 12 | AY027790 | 2p22-p21 | Regulation of apoptosis | -6,23 | -1,00 | -2,13 | -0,75 |
| CASP8 | caspase 8, apoptosis-related cysteine peptidase | NM_001228 | 2q33-q34 | Apoptosis | -5,08 | -4,88 | -0,56 | -0,30 |
| RBM14 | RNA binding motif protein 14 | AF315633 | 11q13.2 | RNA processing and splicing; DNA repair | -5,06 | -4,53 | -1,17 | -1,08 |
| G0S2 | G0/G1switch 2 | NM_015714 | 1q32.2-q41 | Cell cycle regulation | -5,03 | 0,08 | -4,20 | -5,60 |
| ZNFN1A1 | Zinc finger protein, subfamily 1A, 1 (Ikaros) | S80876 | 7p13-p11.1 | Transcription factor | -5,63 | -6,16 | -4,81 | -0,64 |
| BTG2 | BTG family, member 2 | BG339064 | 1q32 | Transcription factor; DNA repair; negative regulation of cell proliferation | -4,81 | -0,60 | -4,66 | -0,69 |
| ***Immune function*** | | | | | | | | |
| TAGAP | T-cell activation GTPase activating protein | NM_138810 | 6q25.3 | Regulatory role in T cell activation | -5,65 | -5,22 | -5,00 | -4,55 |
| JUN | v-jun sarcoma virus 17 oncogene homolog | NM_002228 | 1p32-p31 | T and B cell receptors signalling pathways; MAPK, WNT, TLR signalling pathways | -5,17 | -4,41 | -5,03 | -5,20 |
| RASA3 | RAS p21 protein activator 3 | NM_007368 | 13q34 | Development, adhesion and migration of haemopoietic lineages; GTPase activating protein targeting the ras oncogene | -5,00 | -4,77 | -4,28 | -4,33 |
| TCRα | T cell receptor alpha locus | AE000659 | 14q11.2 | Binding of the complex MHC-peptide; T cell activation | -6,19 | -5,79 | -5,11 | -4,76 |
| IKBKB | Inhibitor of kappa light polypeptide gene enhancer in B-cells, kinase beta | BC006231 | 8p11.2 | T and B cell receptors signalling pathways | -5,52 | -1,97 | -1,86 | -1,74 |
| SLAMF8 | SLAM family member 8 | NM_020125 | 1q23.2 | Migration and ROS production by phagocytes; cell surface receptor | -4,88 | 0,00 | -0,69 | 0,00 |
| CLEC2D | C-type lectin superfamily 2, member D | AW268886 | 12p13 | Surface receptor of NK cells | -5,49 | -6,19 | -2,21 | 0,22 |
| CD300LB | CD300 antigen like family member B | AF427618 | 17q25.1 | Engulfment of apoptotic cells by phagocytes | -5,28 | -1,40 | -5,37 | -1,58 |
| CD6 | CD6 antigen | U66145 | 11q13 | T cell activation; cell adhesion molecules (CAM) | -4,91 | -5,02 | -4,82 | -1,55 |
| ***Signal transduction and transport*** | | | | | | | | |
| TXNDC | thioredoxin domain containing | NM_030755 | 14q22.1 | Signal transduction: response to oxidative stress | -4,98 | -4,79 | -4,69 | -4,81 |
| KCNE3 | Potassium voltage-gated channel, Isk-related family, member 3 | AF302494 | 11q13-q14 | Voltage-gated potassium channel activity | -5,30 | -0,62 | -1,09 | -3,29 |
| APOL6 | Apolipoprotein L6 | AW026509 | 22q12.3 | Lipid transport; lipoprotein metabolism | -5,53 | -5,24 | -0,58 | -0,19 |
| TRPV2 | Transient receptor potential cation channel, subfamily V, member 2 | BF058747 | 17p11.2 | Calcium channel; sensory perception (heat) | -4,93 | -1,56 | -1,26 | -1,69 |
| INPP4A | Inositol polyphosphate-4-phosphatase, type I | NM_001566 | 2q11.2 | Phosphatidylinositol signalling; glycerophospholipid metabolism | -4,68 | -1,06 | -1,54 | -4,59 |
| CLPTM1 | Cleft lip and palate associated transmembrane protein 1 | AF064771 | 12q13.3 | Phosphatidylinositol signalling; glycerophospholipid metabolism | -4,86 | -5,53 | -5,24 | -4,94 |
| PAFAH1B1 | Platelet-activating factor acetylhydrolase, isoform Ib, alpha subunit 45kDa | L13387 | 17p13.3 | Phosphatidylinositol signalling; glycerophospholipid metabolism | -5,14 | -5,02 | -4,63 | -4,67 |
| ***Cytoskeleton; cell-cell and cell-matrix interactions; molecular trafficking; protein folding and ubiquitination*** | | | | | | | | |
| RAB5A | RAB5A, member RAS oncogene family | NM_004162 | 3p24-p22 | Endocytosis; early endosome; small GTPase-mediated signal transduction | -4,90 | -5,01 | -4,74 | -4,54 |
| GNA13 | Guanine nucleotide binding protein (G protein), alpha 13 | NM_006572 | 17q24.3 | Regulation of actin cytoskeleton | -4,85 | -4,65 | -4,69 | -4,46 |
| VIL2 | Villin 2 (ezrin) | BF663141 | 6q25.2-q26 | Cytoskeleton | -5,03 | -5,53 | -4,93 | -5,29 |
| ACTR2 | ARP2 actin-related protein 2 homolog (yeast) | BU175810 | 2p14 | Cytoskeleton | -5,29 | -5,41 | -4,30 | -4,34 |
| DCTN5 | Dynactin 5 (p25) | AI038068 | 16p12.1 | Cytoskeleton | -5,25 | -4,90 | -1,19 | -1,20 |
| HSPA4 | Heat shock 70kDa protein 4 | BC002526 | 5q31.1-q31.2 | Response to unfolded proteins | -5,28 | -5,00 | -4,98 | -5,03 |
| UBE2D3 | Ubiquitin-conjugating enzyme E2D 3 | AL110175 | 4q24 | Ubiquitin-mediated proteolysis | -4,83 | -5,00 | -4,79 | -4,82 |
| GGA2 | Golgi associated gamma adaptin | BC000284 | 16p12 | Transport between the trans-Golgi network and endosomes | -4,81 | -4,68 | -4,86 | -4,90 |
| STIP1 | Stress-induced-phosphoprotein 1 (Hsp70/Hsp90-organizing protein) | AL553320 | 11q13 | Stress-induced chaperone of the Golgi apparatus; over expressed in tumour-infiltrating lymphocytes and macrophages | -4,88 | -4,77 | -4,45 | -4,48 |
| ARFRP1 | ADP-ribosylation factor related protein 1 | AL121845 | 20q13.3 | Intracellular protein transport (Golgi); small GTPase mediated signal transduction | -5,03 | -4,95 | -4,62 | -4,64 |
| MS4A1 | Membrane-spanning 4-domains, subfamily A, member 1 | NM_006544 | 14q22.3 | Post-Golgi transport; protein docking; exocytosis | -4,95 | -5,18 | -4,40 | -4,34 |
| SEC61A1 | Sec61 alpha 1 subunit (S. cerevisiae) | AF346602 | 3q21.3 | Protein export translocase; membrane of the endoplasmatic reticulum | -5,25 | -4,75 | -4,72 | -4,92 |
| SRPRB | Signal recognition particle receptor, B subunit | BF983948 | 3q22.1 | Signal recognition particle; transfer of nascent proteins to endoplasmatic reticulum | -5,06 | -4,95 | -4,87 | -4,80 |
| SENP7 | SUMO1/sentrin specific peptidase 7 | NM_020654 | 3q12 | Proteolysis and peptidolysis; ubiquitin cycle | -5,56 | -5,68 | -5,72 | -1,71 |
| FKBP5 | FK506 binding protein 5 | NM_004117 | 6p21.3-21.2 | Protein folding; unfolded protein binding (chaperone); glucocorticoid signalling | -4,90 | -4,46 | -4,60 | -0,34 |
| WDR42A | WD repeat domain 42A | AA707411 | 1q22-q23 | Nucleocytoplasmic shuttling protein | -5,90 | -2,48 | -4,84 | -1,89 |
| SAR1a | SAR1a gene homolog 1 (*S. cerevisiae*) | BC003658 | 10q22.1 | Intracellular protein transport (endoplasmatic reticulum to Golgi); small GTPase mediated signal transduction | -5,41 | -4,47 | -0,61 | -1,08 |
| ARHGEF7 | Rho guanine nucleotide exchange factor (GEF) 7 | AI040887 |  | Regulation of actin cytoskeleton | -6,14 | -6,56 | -3,93 | -2,12 |
| CTNNA1 | Catenin (cadherin-associated protein), alpha 1, 102kDa | BG330076 | 5q31 | Adherence junction; tight junction | -5,24 | -1,39 | -5,02 | -5,06 |
| SEC8L1 | SEC8-like 1 | AI964022 | 7q31 | Tight junction | -5,17 | -2,24 | -1,49 | -1,37 |
| LAMP1 | Lysosomal-associated membrane protein 1 | J03263 | 13q34 | Lysosome function | -6,26 | -2,75 | -2,71 | -6,00 |
| GOPC | Golgi associated PDZ and coiled-coil motif containing | NM_020399 | 6q21 | Trans-Golgi network; regulation of protein trafficking | -4,93 | -1,41 | -1,50 | -1,33 |
| ***Others*** | | | | | | | | |
| ABCF2 | ATP-binding cassette, sub-family F, member 2 | NM_005692 | 7q36 | Energy metabolism: ATPase activity, mitochondrial membrane | -4,94 | -4,64 | -4,86 | -5,09 |
| PRPS1 | Phosphoribosyl pyrophosphate synthetase 1 | NM_002764 | Xq21-q27 | Energy metabolism: pentose phosphate pathway; purine and histidine metabolism | -6,02 | -6,31 | -5,54 | -5,85 |
| SDHC | Succinate dehydrogenase complex, subunit C, integral membrane protein | AF080579 | 1q21 | Energy metabolism: citrate cycle (tri-carboxylic acid cycle); oxydative phosphorylation | -4,79 | -4,69 | -4,42 | -4,39 |
| GLS | Glutaminase | AI828035 | 2q32-q34 | Energy metabolism: glutamine metabolism to generate ATP and lactate, mitochondrion | -5,18 | -5,22 | -4,73 | -0,37 |
| NADK | NAD kinase | BE674658 | 1p36.33-p36.21 | Nicotinate and nicotinamide metabolism | -5,10 | -1,08 | -4,86 | -1,35 |
| TPD52 | Tumor protein D52 | BG389015 | 8q21 | Regulation of lipid storage | -4,85 | -5,29 | -4,66 | -5,47 |
| ACP5 | Acid phosphatase 5 | NM_001611 | 19p13.3-p13.2 | Riboflavin metabolism | -4,97 | -4,40 | -4,74 | -5,12 |
| ST6GAL1 | ST6 beta-galactosamide alpha-2,6-sialyltranferase 1 | AV695711 | 3q27-q28 | N-glycan biosynthesis | -5,54 | -5,82 | -4,94 | -4,67 |
| PEX16 | peroxisomal biogenesis factor 16 | AB016531 | 11p11.2 | Peroxisome organization and biogenesis | -5,06 | -5,03 | -1,35 | -5,01 |

**Supplementary Table 3. Annotated differentially expressed genes (DEG) belonging to cluster 6 (genes up-regulated in cc2).** Only genes that present a log_2_ ratio (calculated using cc5 as reference) >4 are shown in the table. Relative expressions with cc1 (treated celiac disease patients) and cc8 (untreated celiac disease patient) are also shown, always as log_2_ values. Details for each gene are given on the basis of their Gene Ontology annotation. MSY: male-specific region of Y chromosome.

| **Symbol** | | **Description** | **GenBank** | | **Cytoband** | **Details** | **C2/C5** | **C2/C1** | **C2/C8** |
| --- | --- | --- | --- | --- | --- | --- | --- | --- | --- |
| ***Genes mapping in the region Yq11.2: MSY*** | | | | | | | | | |
| RPS4Y2 | Ribosomal protein S4, Y-linked 2 | | NM_001008 | | Yq11.223 (MSY region) | Ribosomal protein | 10,27 | 10,29 | 10,40 |
| EIF1AY | Eukaryotic translation initiation factor 1A, Y-linked | | BC005248 | | Yq11.223 (MSY region) | Translation initiation factor | 7,66 | 7,66 | 7,69 |
| CYorf15A | Chromosome Y open reading frame 15A | | AF332224 | | Yq11.222-q11.223 (MSY region) | Degenerate gene homologous to the corresponding X-linked CXorf15 and of ancient autosomal genes | 6,95 | 7,02 | 6,94 |
| CYorf15B | Chromosome Y open reading frame 15 B | | AF332225 | | Yq11.222 (MSY region) | Degenerate gene homologous to the corresponding X-linked CXorf15 and of ancient autosomal genes | 6,45 | 6,47 | 6,48 |
| SMCY | Smcy homolog, Y-linked (mouse), lysine demethylase 5D | | NM_004653 | | Yq11.223 (MSY region) | Cell surface receptor: pattern recognition receptor minor histocompatibility antigen; associated to recurrent miscarriages | 7,61 | 7,63 | 7,63 |
| DDX3Y | DEAD (Asp-Glu-Ala-Asp) box polypeptide 3, Y-linked | | NM_004660 | | Yq11.221 (MSY region) | RNA and miRNA processing | 8,10 | 8,05 | 8,07 |
| USP9Y | Ubiquitin specific peptidase 9, Y-linked (fat facets-like, Drosophila) | | AV681765 | | Yq11.221 (MSY region) | Ubiquitination; regulation of protein turnover | 6,19 | 6,27 | 6,30 |
| ***Others*** | | | | | | | | | |
| MARCO | | Macrophage receptor with collagenous structure | | NM_006770 | 2q12-q13 | Scavenger receptor activity | 4,91 | 4,14 | 4,64 |
| RHOQ | | Ras homolog gene family, member Q | | BC033251 | 2p21 | Insulin signalling pathway | 5,34 | 4,78 | 5,15 |
| THBS1 | | Thrombospondin 1 | | AW956580 | 15q15 | Platelet aggregation | 4,82 | 4,88 | 3,84 |

**Supplementary Table 4. Annotated differentially expressed genes (DEG) belonging to cluster 9 (genes up-regulated in cc8, the untreated celiac disease patients not belonging to the family).** Only genes that present a log_2_ ratio (calculated using cc5 as reference) >4 are shown in the table. Relative expressions in comparison with cc1 (treated celiac disease patient) and cc2 (untreated celiac disease patient) are also shown, always as log_2_ values. Details for each gene are given on the basis of their Gene Ontology annotation.

| **Symbol** | **Description** | **GenBank** | **Cytoband** | **Details** | **C8/C5** | **C8/C1** | **C8/C2** |
| --- | --- | --- | --- | --- | --- | --- | --- |
| ***Cell cycle regulators, transcription factors, regulators of gene expression*** | | | | | | | |
| DAB2 | Disabled homolog 2, mitogen-responsive phosphoprotein (*Drosophila*) | BC003064 | 5p13 | Control of cell proliferation | 4,91 | 5,33 | 1,65 |
| CTDSPL | CTD (carboxy-terminal domain, RNA polymerase II, polypeptide A) small phosphatase-like | NM_005808 | 3p21.3 | Transcriptional regulator that silences neuronal genes | 5,89 | 5,46 | 1,59 |
| MEIS1 | Myeloid ecotropic viral integration site 1 homolog (mouse) | NM_002398 | 2p14-p13 | Transcription factor | 4,98 | 5,38 | 5,33 |
| EGF | Epidermal growth factor | NM_001963 | 4q25 | MAPK signalling; cell cycle; cell activation also in response to cytokines | 6,34 | 6,24 | 6,53 |
| TAL1 | T-cell acute lymphocytic leukemia 1 | NM_003189 | 1p32 | Regulation of transcription; cell activation and differentiation | 5,96 | 2,43 | 2,21 |
| CDC14B | CDC14 cell division cycle 14 homolog B (*S. cerevisiae*) | NM_003671 | 9q22.33 | Cell cycle (late nuclear division) | 5,45 | 2,50 | 2,34 |
| DDX11 | DEAD/H (Asp-Glu-Ala-Asp/His) box polypeptide 11 (CHL1-like helicase homolog, *S. cerevisiae*) | NM_030653 | 12p11 | Helicase; maintenance of genomic stability and cohesion of chromosome arms | 4,84 | 0,90 | 0,85 |
| HIST1H4H | Histone 1, H4h | NM_003543 | 6p21.3 | Part of the nucleosome, the basic repeat of eukaryotic chromatic. Epigenetic regulation through methylation | 5,97 | 6,03 | 5,99 |
| HIST1H2AG | Histone gene cluster 1, H2A histone family, member G | NM_021064 | 6p22.1 | Part of the nucleosome, the basic repeat of eukaryotic chromatic. Epigenetic regulation through methylation | 5,98 | 4,94 | 5,45 |
| HIST1H2BG | Histone 1, H2bg | BC001131 | 6p21.3 | Part of the nucleosome, the basic repeat of eukaryotic chromatic. Epigenetic regulation through methylation | 5,26 | 5,63 | 4,84 |
| HIST1H2BC | Histone 1, H2bc | NM_021052 | 1q44 | Part of the nucleosome, the basic repeat of eukaryotic chromatic. Epigenetic regulation through methylation | 6,02 | 6,02 | 5,69 |
| HIST1H2AE | Histone 1, H2ae | AL080170 | 4q12-q13 | Part of the nucleosome, the basic repeat of eukaryotic chromatic. Epigenetic regulation through methylation | 6,14 | 5,96 | 5,67 |
| TRIM58 | Tripartite motif-containing 58 | AL080170 | 1q44 | Cell division; abnormally methylated in carcinomas | 5,03 | 5,53 | 5,18 |
| GAS2L1 | Growth arrest-specific 2 like 1 | BC001782 | 22q12.2 | Cell cycle arrest | 5,20 | 4,94 | 4,92 |
| LAPTM4B | Lysosomal associated protein transmembrane 4 beta | T15777 | 8q22.1 | Cell growth and malignant transformation in many carcinomas | 4,91 | 0,90 | 1,06 |
| MLH3 | mutL homolog 3 (E. coli) | AC006530 | 14q24.3 | Mismatch repair | 4,91 | 5,20 | 5,11 |
| VEPH1 | Ventricular zone expressed PH domain homolog 1 (zebrafish) | AK022666 | 3q24-q25 | Inhibition of TGF-beta induced transcriptional responses | 5,97 | 6,02 | 6,03 |
| MBNL1 | Muscleblind-like (Drosophila) |  |  | RNA binding: splicing regulator | 5,67 | 5,67 | 5,40 |
| ***Immune function and coagulation cascade*** | | | | | | | |
| CEACAM8 | Carcinoembryonic antigen-related cell adhesion molecule 8 | M33326 | 19q13.2 | Marker of granulocyte activation | 6,49 | 7,37 | 7,37 |
| DEFA4 | Defensin, alpha 4, corticostatin | NM_001925 | 8p23 | Antimicrobial activities; mast cell degranulation | 6,60 | 7,58 | 1,99 |
| SCGB1C1 | Secretoglobin, family 1C, member 1 | NM_145651 | 11p15.5 | Immune response in mucosal tissues and fluids | 6,73 | 6,74 | 6,51 |
| MPO | myeloperoxidase | NM_000250 | 17q23.1 | Monocyte function; prostaglandin and leukotriene metabolism | 4,85 | 4,84 | 3,90 |
| BPI | Bactericidal/permeability-increasing protein | NM_001725 | 20q11.23-q12 | Defence against Gram-negative bacteria | 5,10 | 5,61 | 4,58 |
| SELP (CD62) | Selectin P | NM_003005 | 1q22-q25 | Cell adhesion molecule (CAM): interaction of activated endothelial cells/platelets with leukocytes | 6,01 | 5,58 | 5,75 |
| BMP6 | Bone morphogenetic protein 6 | NM_001718 | 6p24-p23 | Inflammatory response | 6,31 | 6,04 | 6,36 |
| ALOX12 | Arachidonate 12-lipoxygenase | NM_000697 | 17p13.1 | Inflammatory response: production of inflammatory metabolites (prostaglandins and leukotrienes) | 5,88 | 2,56 | 2,13 |
| PTGS1 | Prostaglandin-endoperoxide synthase 1 (prostaglandin G/H synthase and cyclooxygenase) | NM_000962 | 9q32-q33.3 | Inflammation: prostaglandin and leukotriene metabolism | 5,23 | 4,97 | 4,94 |
| HPGD | Hydroxyprostaglandin dehydrogenase 15-(NAD) | J05594 | 4q34-q35 | Inflammation: prostaglandin and leukotriene metabolism | 5,00 | 4,16 | 4,23 |
| LCN2 | Lipocalin 2 (oncogene 24p3) | NM_005564 | 9q34 | Associated with neutrophil gelatinase: binding of bacterial LPS and modulation of inflammation | 5,87 | 6,41 | 2,03 |
| TNFSF4 | Tumor necrosis factor (ligand) superfamily, member 4 | NM_003326 | 1q25 | Cytokine-receptor interaction; T cell activation marker | 5,75 | 5,21 | 6,16 |
| CXCL5 | Chemokine (C-X-C motif) ligand 5 | BG166705 | 4q12-q13 | Inflammatory chemokine | 5,05 | 5,11 | 5,11 |
| CKLFSF5 | Chemokine-like factor superfamily 5 | AI147740 | 14q11.2 | Chemotaxis | 5,04 | 4,81 | 4,81 |
| MPL | Myeloproliferative leukemia virus oncogene | NM_005373 | 1p34 | Receptor for thrombopoietin (growth factor for multipotent hematopoietic progenitor cells) | 5,68 | 5,86 | 5,76 |
| GFI1B | Growth factor independent 1B | NM_004188 | 9q34.13 | Regulation of hematopoiesis and megakaryopoiesis | 5,03 | 4,97 | 1,58 |
| HEMGN | Hemogen | AF130060 | 9q22.33 | Differentiation of haematopoietic precursors cells | 4,82 | 5,01 | 5,53 |
| OLFM4 | Olfactomedin 4 | AL390736 | 13q14.3 | Induces differentiation of granulocytes | 6,42 | 6,53 | 6,24 |
| PTCRA | Pre T-cell antigen receptor alpha | AL035587 | 6p21.3 | Precursor of the alpha chain of T-cell receptor | 4,96 | 4,91 | 4,71 |
| CLEC1B | C-type lectin domain family 1, member B | NM_016509 | 12p13.31 | Lectin-like receptor expressed by myeloid and NK cells | 5,64 | 1,93 | 2,09 |
| F2R | Coagulation factor II (thrombin) receptor | BG026194 | 5q13 | Coagulation cascade | 4,89 | 4,72 | 4,82 |
| GP5 | Glycoprotein V (platelet) | NM_004488 | 3q29 | Part of the heterodimeric receptor for von Willebrand factor | 5,08 | 5,58 | 4,65 |
| GP9 | Glycoprotein IX (platelet) | NM_000174 | 3q21 | Part of the heterodimeric receptor for von Willebrand factor | 5,95 | 5,95 | 5,84 |
| GP1BA | Glycoprotein I B (platelet) | NM_000173 | 17pter-p12 | Part of the heterodimeric receptor for von Willebrand factor | 4,84 | 5,04 | 1,49 |
| PROS1 | protein S (alpha) | NM_000313 | 3q11.2 | Coagulation cascade: inhibition of blood clotting | 6,80 | 6,35 | 5,88 |
| ITGB3 | Integrin, beta 3 (platelet glycoprotein IIIa, antigen CD61) | AI189839 | 17q21.32 | Platelet activation; focal adhesion and interactions of platelets with extracellular matrix | 6,30 | 6,19 | 6,32 |
| ITGA2B | Integrin, alpha 2b (platelet glycoprotein IIb of IIb/IIIa complex, antigen CD41B) | AF098114 | 17q21.32 | Platelet activation; focal adhesion and interactions of platelets with extracellular matrix | 5,40 | 5,67 | 5,05 |
| TFPI | Tissue factor pathway inhibitor (lipoprotein-associated coagulation inhibitor) | BF511231 | 2q31-q32.1 | Coagulation cascade | 6,16 | 6,19 | 6,33 |
| CLU | Clusterin (, SP-40,40, sulfated glycoprotein 2, testosterone-repressed prostate message 2, apolipoprotein J) | AI982754 | 8p21-p12 | Complement cascade: complement lysis inhibitor | 5,44 | 5,26 | 5,32 |
| ***Signal transduction and transport*** | | | | | | | |
| ANXA3 | Annexin A3 | M63310 | 4q13-q22 | Phosphatidylinositol signalling: phospholipase A2 inhibitor | 4,80 | 6,64 | 5,99 |
| DLGAP1 | Discs-large associated protein 1 | BC043357 | 18p11.3 | Ion channel; cell-cell signalling | 6,74 | 6,73 | 6,80 |
| HBG2 | Hemoglobin, gamma G | NM_000184 | 11p15.5 | Oxygen transport | 5,93 | 7,00 | 6,44 |
| GNAZ | Guanine nucleotide binding protein (G protein), alpha z polypeptide | NM_002073 | 22q11.22 | GTPase activity; signal transduction | 5,61 | 5,50 | 5,53 |
| GUCY1B3 | Guanylate cyclase 1, soluble, beta 3 | AF020340 | 4q31.3-q33 | Conversion of GTP to the second messenger cGMP | 5,38 | 5,59 | 5,38 |
| PDE5A | Phosphodiesterase 5A, cGMP-specific | BF221547 | 4q25-q27 | Hydrolysis of cGMP | 5,88 | 6,08 | 6,08 |
| RHOBTB1 | Rho-related BTB domain containing 1 | AB018283 | 10q21.2 | Small GTPase-mediated signal transduction | 5,80 | 5,74 | 5,41 |
| ARHGAP18 | Rho GTPase activating protein 18 | AU158022 | 6q22.33 | Small GTPase-mediated signal transduction | 5,78 | 5,32 | 2,25 |
| TCN1 | Transcobalamin I (vitamin B12 binding protein, R binder family) | NM_001062 | 11q11-q12 | Cobalt ion transport | 6,04 | 1,60 | 1,70 |
| SLC24A3 | Solute carrier family 24 (sodium/potassium/calcium exchanger), member 3 | NM_020689 | 20p13 | Ion transport through plasma membrane | 6,60 | 6,91 | 6,39 |
| CABP5 | Calcium binding protein 5 | NM_019855 | 19q13.33 | Signal transduction | 5,53 | 5,58 | 5,59 |
| TBXA2R | Thromboxane A2 receptor | D38081 | 19p13.3 | Calcium signalling | 6,07 | 5,30 | 5,30 |
| XK | Kell blood group precursor (McLeod phenotype) | NM_021083 | Xp21.1 | Integral to membrane; amino acid transport | 6,30 | 6,28 | 5,96 |
| ARHGAP6 | Rho GTPase activating protein 6 | NM_001174 | Xp22.3 | Actin polimerization; Rho signalling | 5,89 | 5,54 | 2,47 |
| ***Cytoskeleton; cell-cell and cell-matrix interactions; molecular trafficking; protein folding and ubiquitination*** | | | | | | | |
| CTTN | Cortactin | NM_005231 | 11q13 | Regulation of actin cytoskeleton; endocytosis; tight junction | 5,68 | 5,63 | 2,08 |
| THBS1 | thrombospondin 1 | BF055462 | 15q15 | Focal adhesion | 6,30 | 6,26 | 1,69 |
| PARVB | Parvin, beta | AA187563 | 22q13.2-q13.33 | Focal adhesion | 5,48 | 5,02 | 1,77 |
| CAV2 | Caveolin 2 | NM_001233 | 7q31.1 | Focal adhesion | 5,46 | 5,57 | 5,59 |
| CTNNAL1 | catenin (cadherin-associated protein), alpha-like 1 | NM_003798 | 9q31.2 | Cell adhesion; cadherin binding; actin cytoskeleton | 5,68 | 5,28 | 5,33 |
| PLOD2 | Procollagen-lysine, 2-oxoglutarate 5-dioxygenase 2 | NM_000935 | 3q23-q24 | Hydroxylation of lysine residues in collagen; cell migration; TGF pathway | 5,28 | 5,37 | 5,41 |
| LTBP1 | Latent transforming growth factor beta binding protein 1 | NM_000627 | 2p22-p21 | Extracellular matrix; TGF pathway | 5,48 | 6,14 | 5,44 |
| EPB49 | Erythrocyte membrane protein band 4.9 (dematin) | NM_001978 | 8p21.1 | Actin filament bundle formation | 5,44 | 4,68 | 1,20 |
| CALD1 | Caldesmon 1 | AL583520 | 7q33 | Actin and myosin binding; component of cytoskeleton; contraction | 6,42 | 6,98 | 6,15 |
| PHACTR2 | phosphatase and actin regulator 2 | R81072 | 6q24.2 | Actin binding | 7,00 | 6,94 | 6,93 |
| DNM3 | dynamin 3 | AL136712 | 1q24.3 | Actin-membrane processes, predominantly membrane budding and endocytosis | 6,14 | 6,16 | 6,19 |
| MMRN1 | Multimerin 1 | NM_007351 | 4q22 | Cell adhesion | 6,17 | 6,64 | 6,55 |
| DDEF2 | Development and differentiation enhancing factor 2 | NM_003887 | 2p25 | Golgi stack; small GTPase mediated signal transduction | 5,44 | 5,44 | 5,41 |
| FKBP1B | FK506 binding protein 1B | NM_004116 | 2p23.3 | Protein folding | 5,01 | 4,40 | 0,88 |
| PCSK6 | Proprotein convertase subtilisin/kexin type 6 | NM_002570 | 15q26.3 | Endoplasmic reticulum; proteolysis; cell-cell signalling | 6,16 | 6,35 | 6,39 |
| SRP68 | signal recognition particle 68kDa | AFFX-HUMRGE/M10098_3 | 17q25.1 | Targeting of proteins to the endoplasmatic reticulum | 8,41 | 8,84 | 8,67 |
| CRISP3 | Cysteine-rich secretory protein 3 | NM_006061 | 6p12.3 | Extracellular matrix; cell-cell adhesion; granules; innate immunity | 6,86 | 7,07 | 6,97 |
| FSTL1 | Follistatin-like 1 | BC000055 | 3q13.33 | Keratonocyte migration and wound repair, either as mRNA and miRNA | 6,58 | 5,74 | 5,83 |
| CHI3L1 | Chitinase 3-like 1 (cartilage glycoprotein-39) | M80927 | 1q32.1 | Structural constituent of extracellular matrix | 5,01 | 5,13 | 5,20 |
| CEACAM6 | Carcinoembryonic antigen-related cell adhesion molecule 6 (non-specific cross reacting antigen) | M18728 | 19q13.2 | Integral to plasma membrane: cell-cell signalling | 6,37 | 6,45 | 5,62 |
| SPARC | Secreted protein, acidic, cysteine-rich (osteonectin) | AL575922 | 5q31.3-q32 | Matrix-associated, elicits changes in cell shape and influences the synthesis of extracellular matrix | 5,24 | 5,65 | 5,90 |
| JAM3 | Junctional adhesion molecule 3 | AA149644 | 11q25 | Cell adhesion molecule (CAM); tight junction | 6,25 | 5,51 | 6,35 |
| ESAM | endothelial cell adhesion molecule | AL573851 | 11q24.2 | Cell adhesion molecule (CAM); tight junction | 5,52 | 5,39 | 5,45 |
| CTTN | Cortactin | BG475299 | 11q13 | Tight junctions | 7,38 | 7,41 | 7,15 |
| EHD3 | EH-domain containing 3 | NM_014600 | 2p21 | Regulation of endocytic recycling | 5,91 | 5,21 | 5,08 |
| STN2 | Stonin 2 | AA632295 | 14q31.1 | Regulation of endocytosis | 5,69 | 5,76 | 6,14 |
| SYTL4 | Synaptotagmin-like 4 (granuphilin-a) | AL391688 | Xq22.1 | Regulation of exocytosis | 6,34 | 6,39 | 6,39 |
| FNTB | Farnesyltransferase, CAAX box, beta | BF131248 | 14q23-q24 | Attaches a farnesyl group to cys residues of proteins that must be translocated to membranes | 5,17 | 5,32 | 5,49 |
| USP37 | Ubiquitin specific peptidase 37 | AI436136 | 2q35 | Degradation of ubiquitinated proteins | 5,39 | 5,47 | 5,47 |
| ***Others*** | | | | | | | |
| GMPR | Guanosine monophosphate reductase | NM_006877 | 6p23 | Purine metabolism: maintenance of the A/G balance | 5,53 | 5,16 | 5,40 |
| PVALB | Parvalbumin | NM_002854 | 22q12-q13 | Calcium ion binding; contraction; muscle development | 6,05 | 6,17 | 6,17 |
| SMOX | Spermine oxidase | BC000669 | 20p13 | Oxidoreductase activity | 5,72 | 5,68 | 2,58 |
| CA13 | Carbonic anhydrase XIII | BF111998 | 8q21.2 | Reversible hydratation of carbon dioxide. Acid-base balance during respiration, calcification, etc. | 5,20 | 4,77 | 4,77 |
| ATP9A | ATPase, Class II, type 9A | AB014511 | 20q13.11-q13.2 | ATPase activity to translocate phospholipids in the plasma membrane | 5,46 | 5,88 | 6,07 |
| HGD | Homogentisate 1,2-dioxygenase (homogentisate oxidase) | AI478172 | 3q21-q23 | Phenylalanine and tyrosine catabolism | 6,55 | 6,61 | 6,64 |
| ARG2 | Arginase, type II | U75667 | 14q24.1-q24.3 | Urea cycle and metabolism of amino groups | 5,57 | 5,54 | 5,57 |
| BCKDHA | Branched chain keto acid dehydrogenase E1, alpha polypeptide (maple syrup urine disease) | BF446281 | 19q13.1-q13.2 | Aminoacid catabolism: oxidative decarboxylation of branched chains alpha-ketoacids derived from ile, leu, val | 4,82 | 4,42 | 4,50 |
| MYLK | Myosin light polypeptide kinase | AA526844 | 3q21 | Muscle contraction; also expressed in other tissues | 5,45 | 2,18 | 2,05 |
| MYO15B | Myosin XVB pseudogene | BF478120 | 17q25.1 | Transcribed, unprocessed pseudogene | 5,15 | 1,92 | 4,78 |
| MGLL | Monoglyceride lipase | BG168471 | 3q21.3 | Glyceropolid metabolism | 5,09 | 5,06 | 4,94 |
| ANKRD9 | Ankyrin repeat domain 9 | AW194999 | 14q32.32 | Lipid metabolism and intracellular accumulation: upregulated during lipid metabolic perturbations | 5,32 | 5,23 | 5,41 |
| FRMD3 | FERM domain containing 3 | AA746863 | 9q21.32 | Maintenance of erythrocyte shape | 5,03 | 5,03 | 4,74 |

**Supplementary Table 5. Annotated differentially expressed genes (DEG) belonging to cluster 10 (genes down-regulated in cc1, up-regulated in cc2-cc4).** Only genes that present a log_2_ (calculated using cc5 as reference) >4 for the ratio cc2/cc5 or <-4 for the ratio cc1/cc5 are shown in the table. Relative expressions with cc1/cc2, cc1/cc3 and cc1/cc4 are also shown, always as log_2_ values. Details for each gene are given on the basis of their Gene Ontology annotation.

| **Symbol** | **Description** | **GenBank** | **Cytoband** | **Details** | | **C1/C5** | | **C2/C5** | | **C1/C2** | | **C1/C3** | | **C1/C4** |
| --- | --- | --- | --- | --- | --- | --- | --- | --- | --- | --- | --- | --- | --- | --- |
| ***Cell cycle regulators, transcription factors, regulators of gene expression*** | | | | | | | | | | | | | | |
| CUGBP2 | CUG triplet repeat, RNA binding protein 2 | W79537 | 10p13 | Co-transcriptional and post-transcriptional RNA processing | | -1,03 | | 4,80 | | -5,83 | | -6,15 | | -6,32 |
| SFRS6 | Splicing factor, arginine/serine-rich 6 | NM_006275 | 20q12-q13.1 | Nuclear mRNA splicing via spliceosome | | 0,45 | | 6,10 | | -5,66 | | -0,43 | | -1,72 |
| SF1 | Splicing factor 1 | D26121 | 11q13 | Spliceosome assembly | | -1,13 | | 6,42 | | -7,56 | | -6,35 | | -8,23 |
| EIF3S8 | Eukaryotic translation initiation factor 3, subunit 8, 110kDa | AI377875 | 16p11.2 | Regulation of translational initiation | | -0,15 | | 5,55 | | -5,70 | | -2,26 | | -1,60 |
| CLK4 | CDC-like kinase 4 | AA468591 | 5q35 | Delay of cytokinesis in case of damages to chromatin | | -4,29 | | 1,76 | | -6,06 | | -4,83 | | -5,92 |
| BIRC6 | Baculoviral IAP repeat-containing 6 (apollon) | AA608834 | 2p22-p21 | Anti-apoptotic | | -0,22 | | 4,93 | | -5,15 | | -5,37 | | -5,06 |
| IREB2 | Iron-responsive element binding protein 2 | AW470799 | 15q25.1 | Post-transcriptional regulation of mRNA stability: stabilization of mRNAs containing the IRE motifs in their 3’ UTR | | -1,51 | | 4,86 | | -6,37 | | -5,16 | | -5,92 |
| HNRPD | Heterogeneous nuclear ribonucleoprotein D (AU-rich element RNA binding protein 1, 37kDa) | AA863112 | 4q21.1-q21.2 | Post-transcriptional regulation of mRNA stability: Degradation of mRNAs containing the instability-motifs AURE in their 3’ UTR | | -1,27 | | 4,50 | | -5,77 | | -6,31 | | -6,22 |
| PAN3 | Pab1p-dependent poly(A) nuclease | AI701943 | 13q12.2 | Post-transcriptional regulation of mRNA stability: shortening of mRNA poly(A) tails and mRNA degradation | | -0,69 | | 4,82 | | -5,51 | | -5,70 | | -6,24 |
| RC3H1 | Ring finger and CCCH-type zinc finger domains 1 | AW205418 | 1q25.1 | Post-transcriptional regulation of mRNA stability: RNA binding protein localized in cytosolic RNA granules | | -0,45 | | 4,79 | | -5,25 | | -3,63 | | -5,68 |
| DDX17 | DEAD (Asp-Glu-Ala-Asp) box polypeptide 17 | AA521056 | 22q13.1 | Regulation of mRNA splicing and stability; cell growth and division | | -1,13 | | 4,26 | | -5,38 | | -4,79 | | -6,43 |
| YAF2 | YY1 associated factor 2 | AA651631 | 12q12 | Regulation of transcription; regulation of apoptosis during genotoxic stress responses | | -0,03 | | 4,84 | | -4,87 | | -4,94 | | -4,46 |
| ZNF198 | Zinc finger protein 198 | AL136621 | 13q11-q12 | Regulation of transcription | | -0,71 | | 4,33 | | -5,04 | | -5,12 | | -5,53 |
| RFX3 | Regulatory factor X, 3 | BE466926 | 9p24.2 | Ciliogenic transcription factor: regulation of cilia genes | | 0,72 | | 4,63 | | -3,91 | | -0,39 | | -4,25 |
| PHF20L1 | PHD finger protein 20-like 1 | AW205964 | 8q24.22 | Regulation of DNA methylation and transcription | | 2,77 | | 4,65 | | -1,88 | | -1,55 | | -2,04 |
| SMARCC1 | SWI/SNF related, matrix associated, actin dependent regulator of chromatin, subfamily c, member 1 | AI208857 | 3p23-p21 | Chromatin remodelling and transcriptional activation | | -0,42 | | 4,29 | | -4,71 | | -4,73 | | -4,80 |
| EML4 | Echinoderm microtubule associated protein like 4 | AA524507 | 2p22-p21 | Organization of mitotic spindle; mitotic progression | | -4,03 | | 0,51 | | -4,55 | | -4,67 | | -5,14 |
| ***Immune function and coagulation cascade*** | | | | | | | | | | | | | | |
| KLF9 | Kruppel-like factor 9 | AI690205 | 9q13 | Quiescence-associated transcription factor for naïve B-cells | | -0,73 | | 4,73 | | -5,46 | | -5,55 | | -5,22 |
| ***Signal transduction and transport*** | | | | | | | | | | | | | | |
| PDE4D | Phosphodiesterase 4D, cAMP-specific (phosphodiesterase E3 dunce homolog, Drosophila) | AI082004 | 5q12 | Production of the second messenger cAMP | | 0,59 | | 5,15 | | -4,56 | | -4,46 | | -0,58 |
| SLC25A36 | Solute carrier family 25, member 36 | AW514168 | 3q23 | Pirimidine transporter of the inner mitochondrial membrane | | -0,89 | | 4,48 | | -5,37 | | -5,49 | | -5,95 |
| ***Cytoskeleton; cell-cell and cell-matrix interactions; molecular trafficking; protein folding and ubiquitination*** | | | | | | | | | | | | | | |
| SSH2 | slingshot homolog 2 (Drosophila) | AA975530 | 17q11.2 | Regulation of actin cytoskeleton | | -5,70 | | 1,07 | | -6,77 | | -7,18 | | -7,00 |
| THBS1 | thrombospondin 1 | AV726673 | 15q15 | Extracellular matrix; focal adhesion | | 0,47 | | 6,02 | | -5,54 | | -5,02 | | -0,21 |
| ATP8A1 | ATPase, aminophospholipid transporter (APLT), Class I, type 8A, member 1 | AI424825 | 4p14-p12 | Endosomal membrane trafficking; endosome recycling | | -0,71 | | 5,07 | | -5,79 | | -5,92 | | -6,80 |
| UBE2D2 | Ubiquitin-conjugating enzyme E2D 2 (UBC4/5 homolog, yeast) | AI949690 | 5q31.2 | Ubiquitin-mediated proteolysis | | -0,73 | | 5,02 | | -5,75 | | -1,97 | | -6,22 |
| SMAP1L | Stromal membrane-associated protein 1-like | T90760 | 1p35.3-p34.1 | Retrograde trafficking of clathrin-coated vescicles between early endosomes and trans-Golgi network | | 0,84 | | 5,26 | | 5,26 | | -4,93 | | -4,33 |
| UBE2D2 | Ubiquitin-conjugating enzyme E2D 2 (UBC4/5 homolog, yeast) | AF116659 | 5q31.2 | Ubiquitin-mediated proteolysis | | -1,13 | | 4,15 | | -5,28 | | -5,00 | | -1,69 |
| ***Others*** | | | | | | | | | | | | | | |
| PSPH | Phosphoserine phosphatase | NM_003832 | 7p15.2-p15.1 | Glycine, serine and threonine metabolism | -0,18 | | 7,92 | | -8,10 | | -7,52 | | -7,33 | |
| SESN3 | Sestrin 3 | BF514585 | 11q21 | Regulation of glucose and lipid metabolism; protection against oxidative stress | -0,42 | | 5,76 | | -6,18 | | -6,42 | | -7,02 | |
